# Supplementary material for: Hierarchically manufactured chiral plasmonic nanostructures with gigantic chirality for polarized emission and information encryption
Source: Nat Commun. 2023 Nov 10;14:7298. doi: 10.1038/s41467-023-43112-6 (PMC10638435; doi:10.1038/s41467-023-43112-6)
Supplement: Supplementary file 1 — Supplementary Information [file 41467_2023_43112_MOESM1_ESM.pdf]

Supplementary Information

Hierarchically manufactured chiral plasmonic nanostructures with gigantic chirality for polarized emission and information encryption

Yoon Ho Lee[1,2,3,8], Yousang Won[1,8], Jungho Mun[4,8], Sanghyuk Lee[1], Yeseul Kim[4], Bongjun Yeom[5], Letian Dou[2], Junsuk Rho[4,6,7,*], Joon Hak Oh[1,*]

[1] School of Chemical and Biological Engineering, Institute of Chemical Processes, Seoul National University, Seoul 08826, Republic of Korea.

[2] Davidson School of Chemical Engineering, Purdue University, West Lafayette, IN 47907, USA

[3] Department of Chemistry, Purdue University, West Lafayette, IN 47907, USA

[4] Department of Mechanical Engineering, Pohang University of Science and Technology (POSTECH), Pohang 37673, Republic of Korea.

[5] Department of Chemical Engineering, Hanyang University, Seoul 04763, Republic of Korea.

[6] Department of Chemical Engineering, Pohang University of Science and Technology (POSTECH), Pohang 37673, Republic of Korea.

[7] POSCO-POSTECH-RIST Convergence Research Center for Flat Optics and Metaphotonics, Pohang 37673, Republic of Korea.

[8] These authors contributed equally: Yoon Ho Lee, Yousang Won, Jungho Mun.

* E-mail: joonhoh@snu.ac.kr, jsrho@postech.ac.kr

Supplementary Note 1.

To investigate the origin of the light absorption peak of the chiral plasmonic structures with nanogratings, we measured absorbance spectra of three types of substrate samples: flat PDMS/Au substrates, PDMS/Au substrates with the original nanogratings, and those with original nanogratings combined with the vertical nanogratings (40-nm-thick), as shown in **Supplementary Fig. 11**. The PDMS with both the original and additionally formed vertical nanogratings was fabricated by stretching the original nanograting-patterned PDMS film perpendicular to the original nanograting direction and then applying UV/O_3 surface treatment. The schematic images of flat, original nanogratings-based, and original with additionally formed vertical nanogratings-based PDMS/Au substrates are presented in **Supplementary Fig. 11a-c**, respectively. The corresponding absorption spectra of these samples are shown in **Supplementary Fig. 11d**. The absorption spectra indicated that flat PDMS/Au predominantly absorbs light in the 300-500 nm wavelength range, confirming that the main absorption peak centered around 400 nm wavelength in our chiral PDMS/Au is due to the intrinsic absorption of Au. Additionally, both PDMS/Au substrate with the original nanogratings and PDMS/Au substrate with the original nanogratings combined with the vertical nanogratings showed increased absorption at 480 nm compared to the flat PDMS/Au substrate. Notably, the PDMS/Au substrate with the original nanogratings combined with the vertical nanogratings showed a higher absorption peak at 480 nm wavelength than the others, along with an additional absorption peak at 630 nm. It means that the additional shoulder peaks around 480 and 630 nm in chiral plasmonic structures with nanogratings are attributed to the nanograting s. The calculated absorption spectra of flat PDMS/Au substrate, PDMS/Au with the original n anogratings (540 nm pitch), and PDMS/Au with the additionally formed nanogratings (100 n m pitch) also exhibited similar trends, consistent with the experimental results of the three typ es of samples (**Supplementary Fig. 12**). In the flat PDMS/Au, light absorption was mainly ob served in the 300-500 nm range, while the PDMS/Au with the additionally formed nanogratin gs (100 nm pitch) showing an additional optical absorption peak at 630 nm wavelength. Furth ermore, all of the PDMS/Au with nanogratings showed stronger light absorption at 480 nm co mpared to the flat PDMS/Au. The heights of the nanogratings were 10 and 20 nm for 540 nm pitch and 100 nm pitch, respectively.

Supplementary Note 2.

We performed a chiroptical analysis of LH- and RH-chiral plasmonic structures with Au coatings of 30, 40, 50 nm, or 130 nm and with 830 nm original nanograting patterns with different pitch size. **Supplementary Fig. 14a** shows the CD spectra of the LH- and RH-patterned chiral plasmonic structures with an Au layer 30, 40, or 50 nm thick. The peak positions of the CD spectra shifted to 504, 511, and 500 nm, respectively. Broader peaks were obtained in the CD spectra of the 40- and 50 nm thick than in those of the 30 nm thick Au-based chiral plasmonic structures while all chiral patterns had similar *g*-factor values (**Supplementary Fig. 14b**). The chiral plasmonic structures with 30 and 50 nm thick Au coatings had high *g*-factor values (both 0.06). The chiral plasmonic patterns covered with 130 nm thick Au showed similar CD spectra but relatively lower values for both CD and g-factor, probably due to the decreased interaction of plasmonic modes at the Au/air and PDMS/Au interfaces (**Supplementary Figs. 15a** and **15b**). The 130 nm thick Au chiral plasmonic films based on the inverted chiral PS film had similar CD spectra, with vertically mirrored positive and negative signals for the LH- and RH-patterned chiral plasmonic structures (**Supplementary Figs. 15c and 15d**). The CD and *g*-factors of the chiral (based on PDMS) and inverted (based on PS) chiral plasmonic structures were determined based on the original grating nanopatterns with 830 nm pitch sizes and 40 nm thick Au (**Supplementary Fig. 16)**. The positions of the CD peaks were around 700 nm and thus significantly red-shifted compared to the chiral plasmonic structures based on the original nanograting pattern with a 540 nm pitch, which had a peak position of 511 nm. These results showed that the peak positions of the CD spectra can be controlled by adjusting the thickness of the metal film and the pitch of a simple nanograting pattern. Note that *g*-factor values > 0.07 at 700 nm were achieved for the inverted chiral plasmonic structures (**Supplementary Fig. 16d**).

a

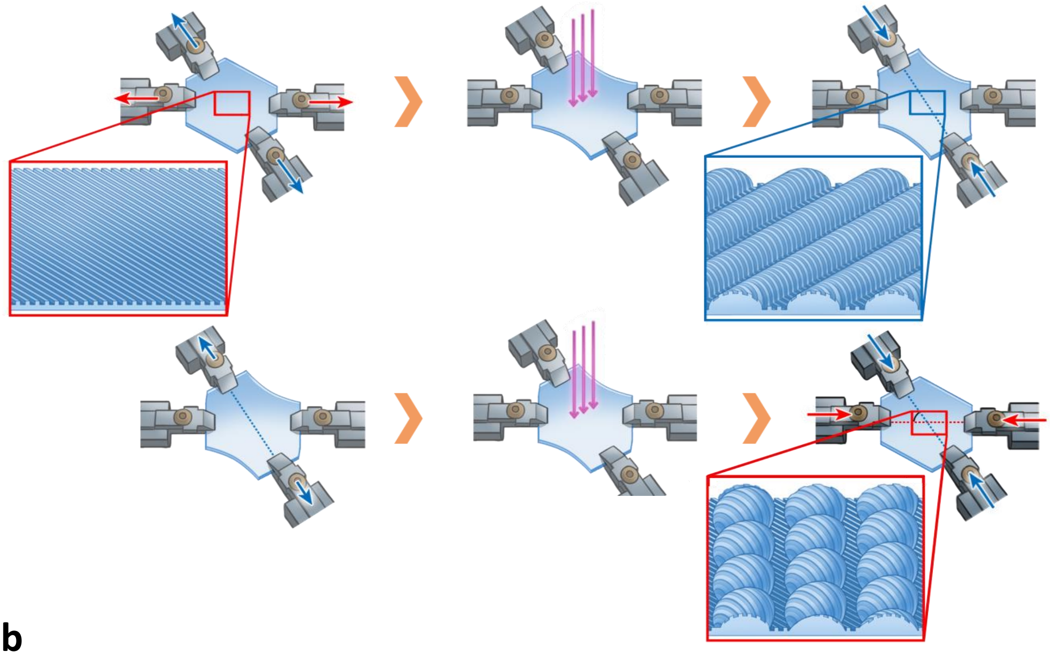

b

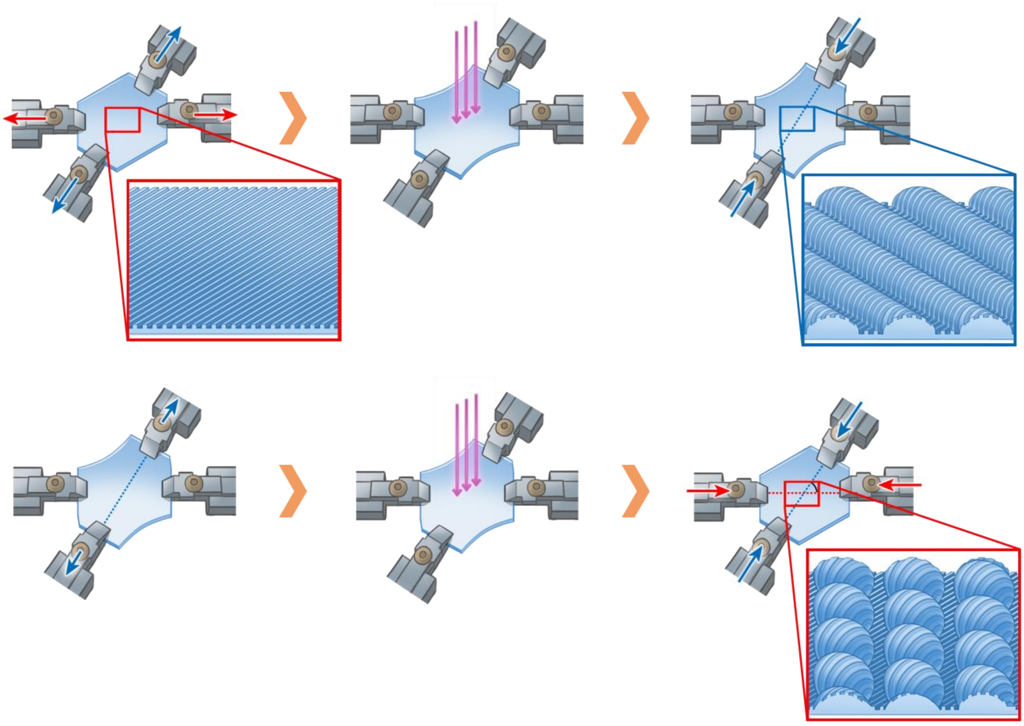

Supplementary Fig. 1| a,**b**, Fabrication processes of the **(a)** LH- and **(b)** RH- chiral structured PDMS substrates.

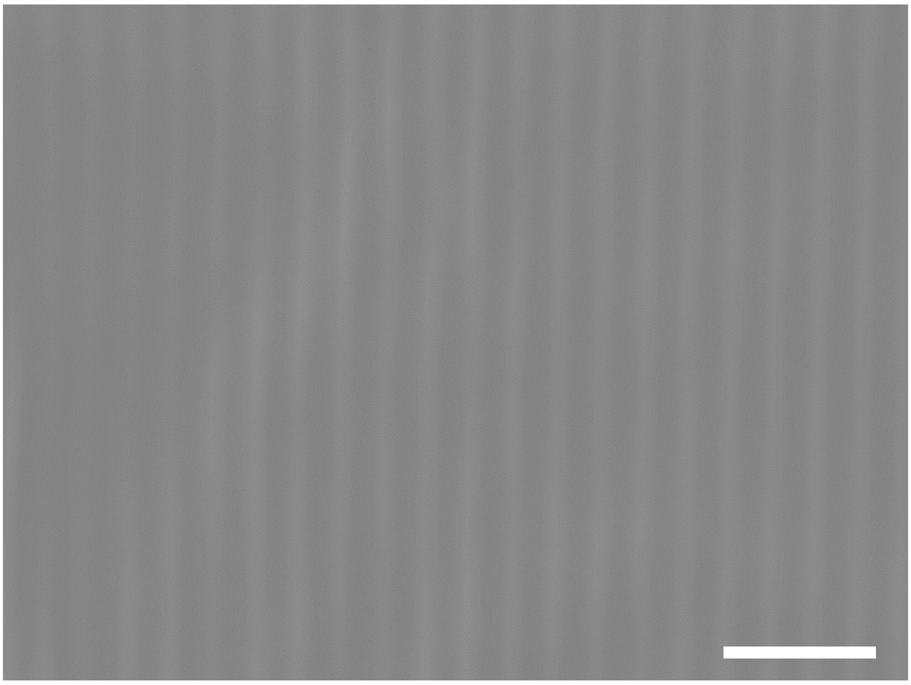

Supplementary Fig. 2| SEM images of 540 nm pitch sized nanograting patterned PDMS/Au (40 nm) substrates (scale bar: 2 μm).

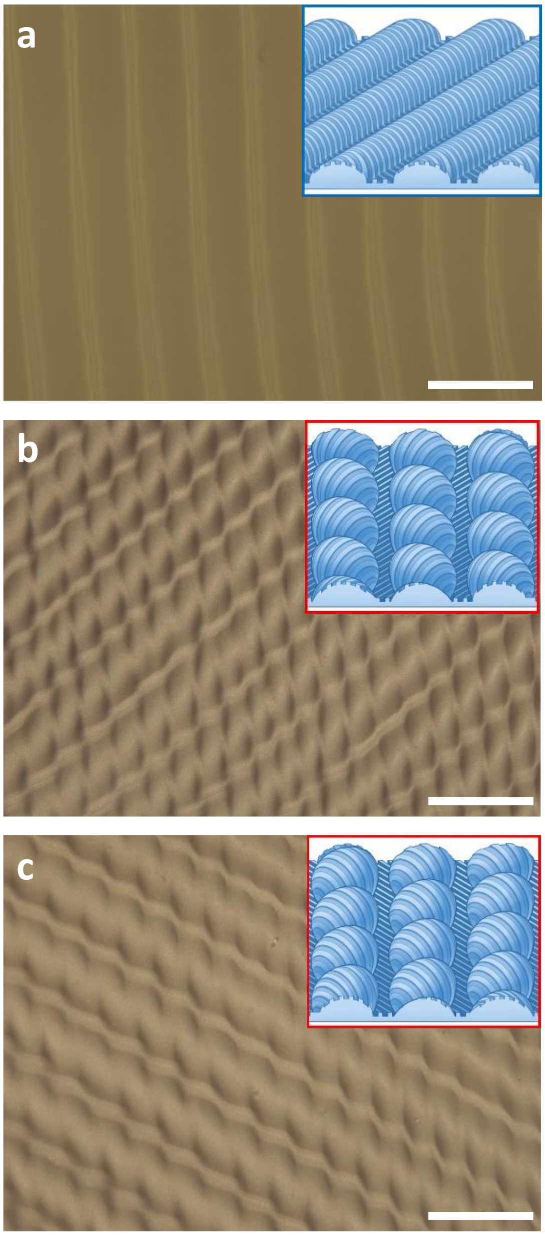

Supplementary Fig. 3| (a) OM image of PDMS after the first stretching and release process. **b,c,** OM images of **(b)** LH- and **(c)** RH-patterned PDMS films (scale bar: 50 μm).

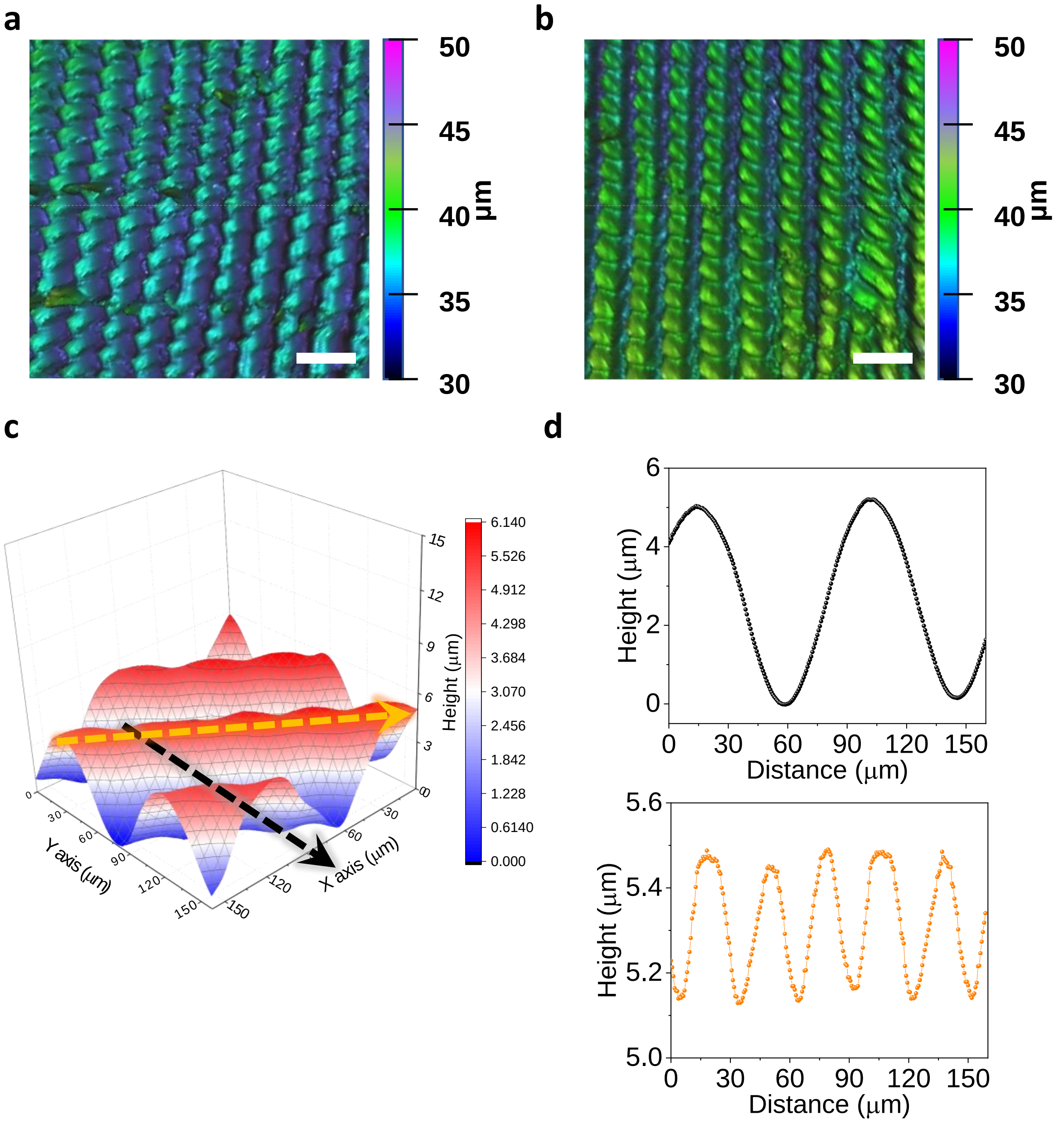

Supplementary Fig. 4| 3D profiler results of chiral plasmonic structures. a, b, 3D profiler images of **(a)** LH- and **(b)** RH-patterned chiral plasmonic structures (scale bar: 100 μm). (**c**) 3D profiler image of the RH-patterned chiral plasmonic structures with *x*, *y*, *z* coordinates. (**d**) Cross-sectional line profiles for the black-dashed (top) and orange-dashed (bottom) lines in Supplementary Fig. 4c.

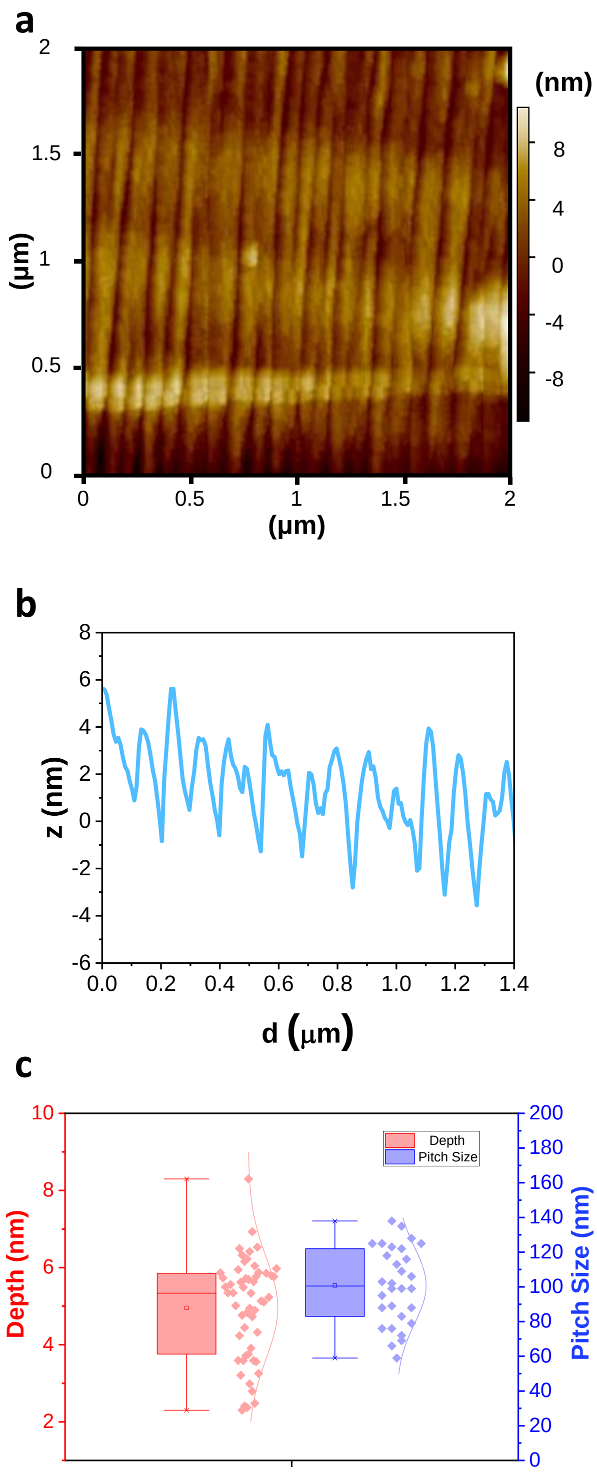

Supplementary Fig. 5| a,b, (a) AFM image and **(b)** height profile of additionally formed diagonal nanopatterns of chiral plasmonic films. **(c)** Pitch and depth distributions of the diagonal nanopatterns.

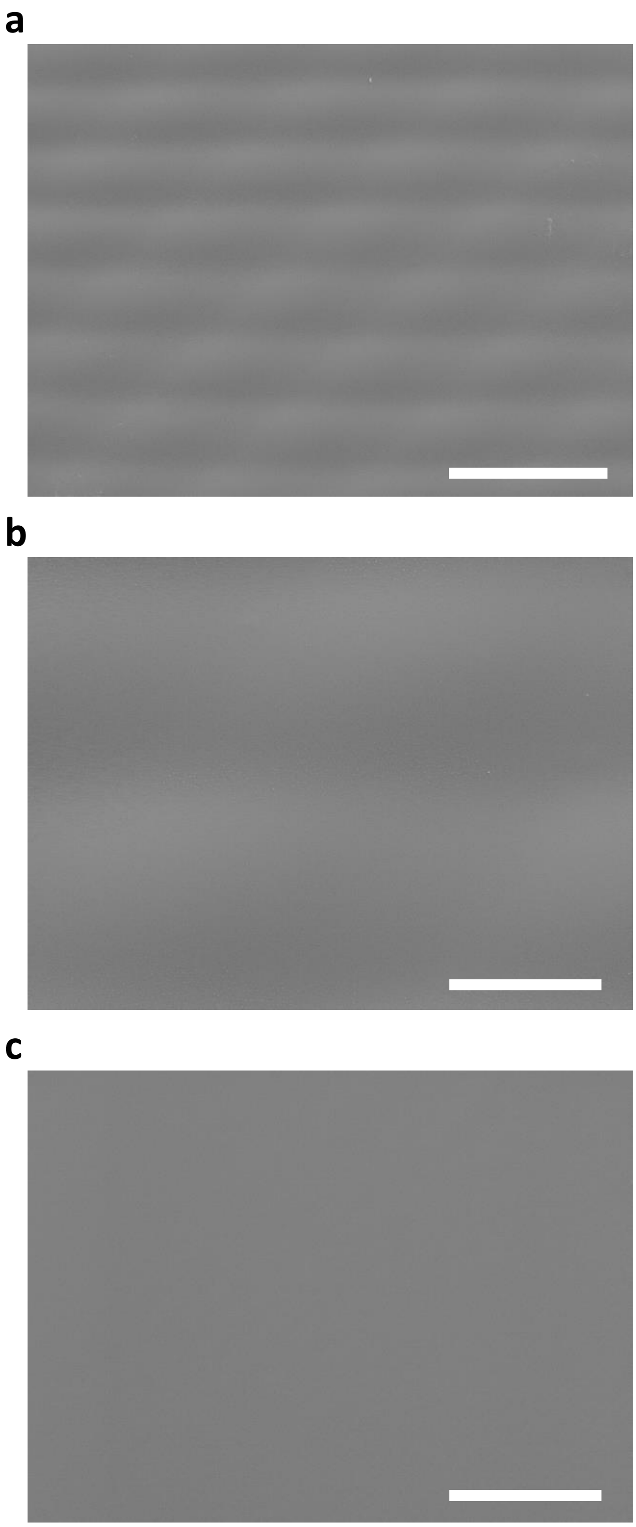

Supplementary Fig. 6| SEM images of LH-patterned chiral plasmonic films without nanograting patterns (scale bars of Supplementary Fig. 6a: 100 µm, 6b: 30 µm, 6c: 3 µm)

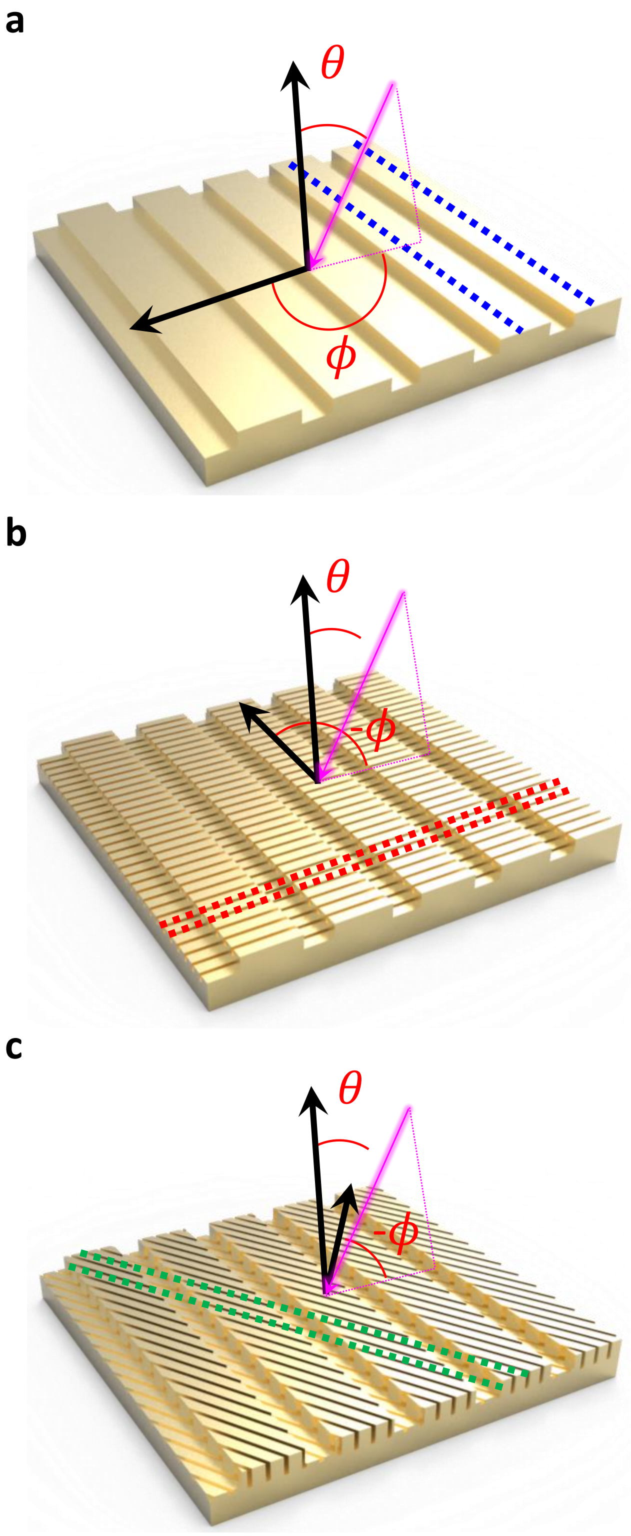

Supplementary Fig. 7| a–c, Schematic images of the **(a)** original grating patterns, the additionally formed **(b)** vertical nanopatterns, and the **(c)** diagonal nanopatterns, including θ and ϕ, for LH-chiral plasmonic patterns (pink arrow: incident light direction).

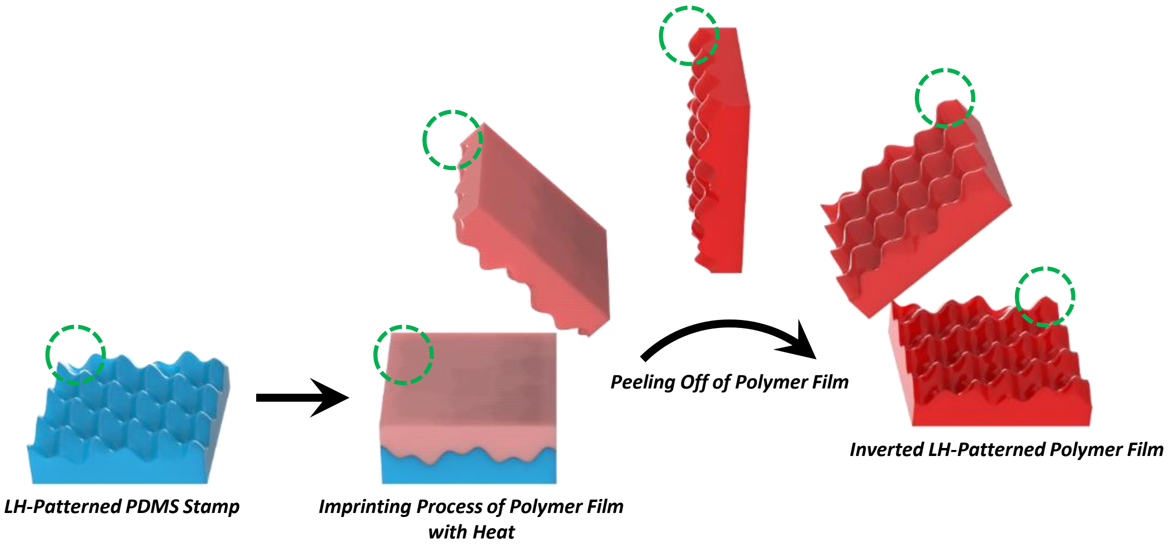

Supplementary Fig. 8| Schematic images of imprinting process for inverted-LH chiral patterned polymer film using LH chiral patterned PDMS stamp. The green dashed circle represents the transformation of the polymer film, located in the concave region of the LH chiral pattern PDMS stamp after nanoimprinting, into a convex area.

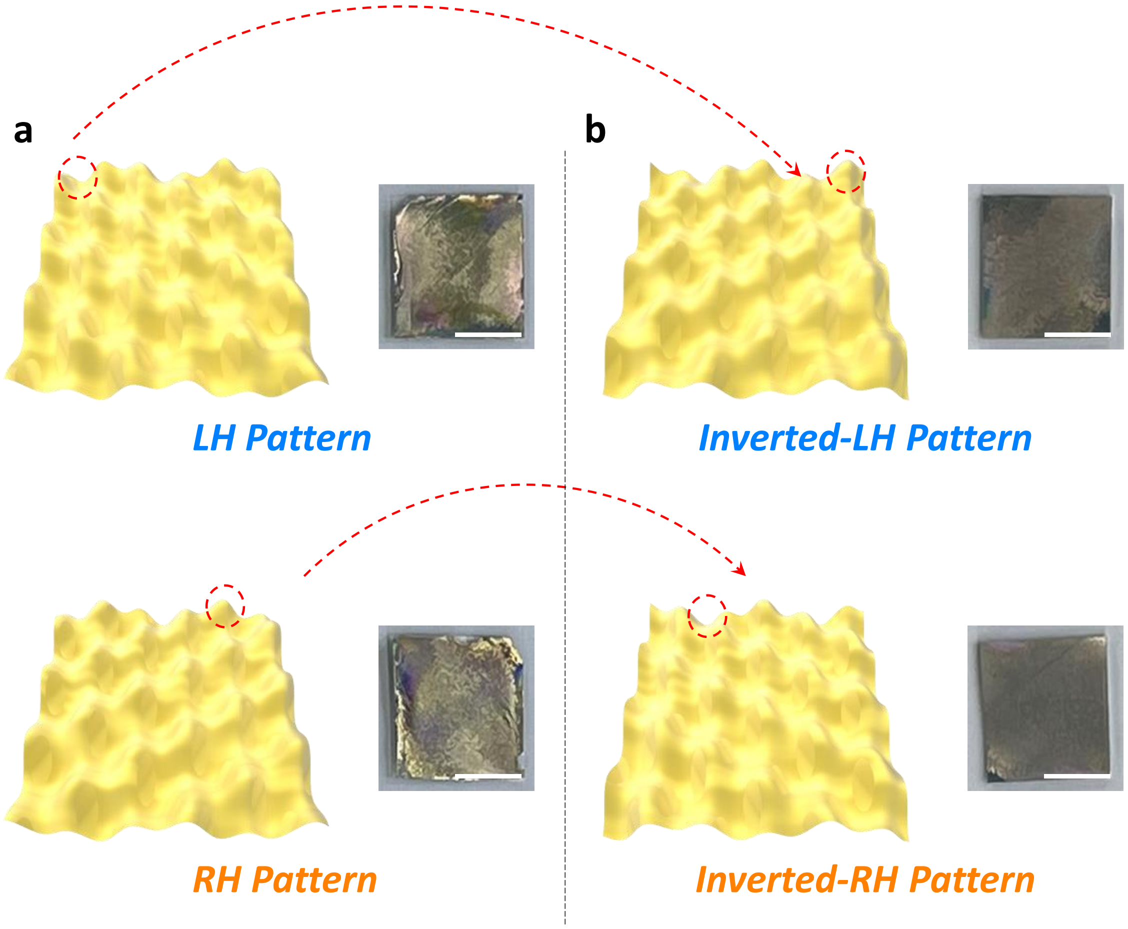

Supplementary Fig. 9| a,**b**, Schematic images (left) with corresponding photograph images (right) of **(a)** LH- and RH-chiral plasmonic patterns (based on PDMS films) and **(b)** inverted LH- and RH-chiral plasmonic patterns (based on PS films) without nanogratings. (red dashed arrows and circles: in the case of inverted patterns, the concave regions of the original pattern are transformed into convex regions, protruding outward, while the convex regions are transformed into concave regions, indenting inward in the opposite direction; scale bars in the photograph images: 5 mm).

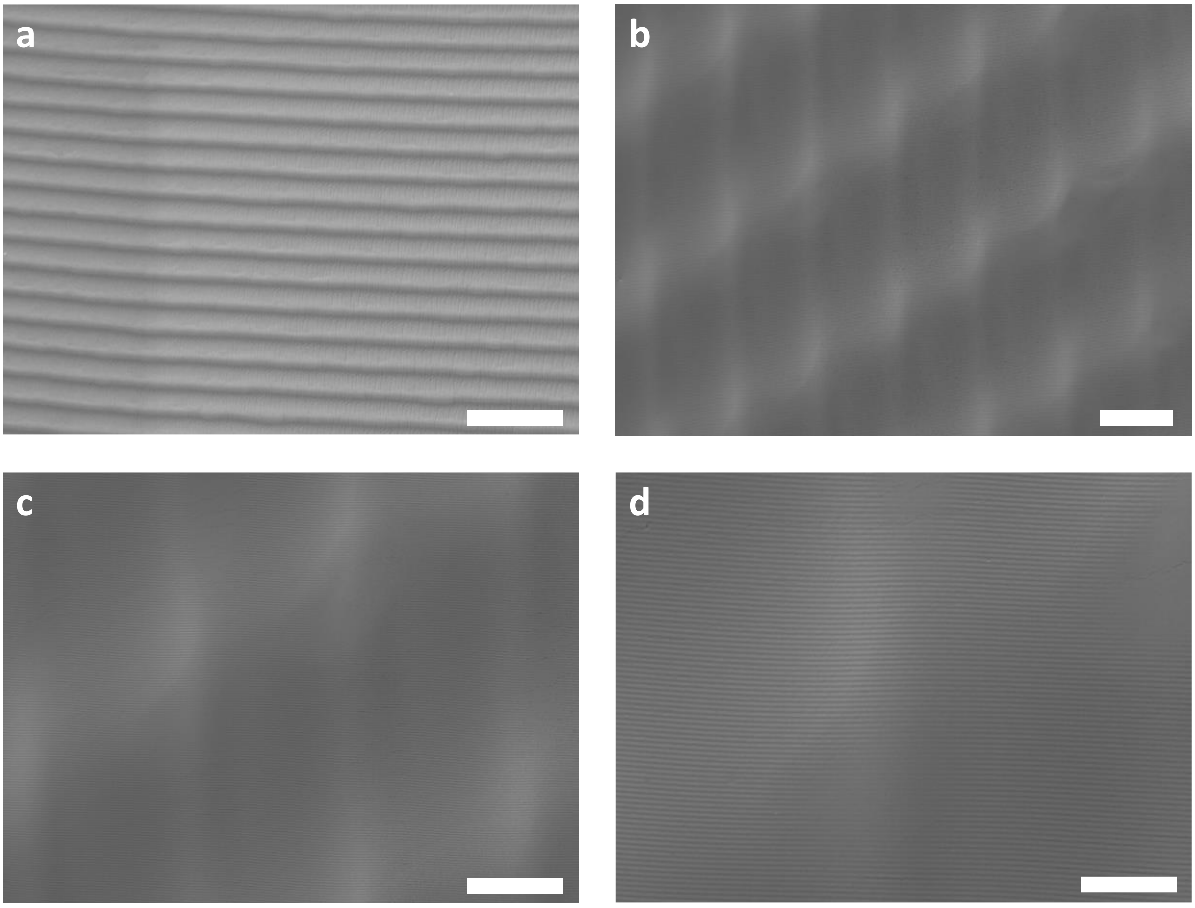

Supplementary Fig. 10| SEM images of inverted LH-patterned plasmonic films based on PS substrates (scale bars of Supplementary Fig. 10a: 2 μm, 10b: 30 μm, 10c: 10 μm, 10d: 20 μm)

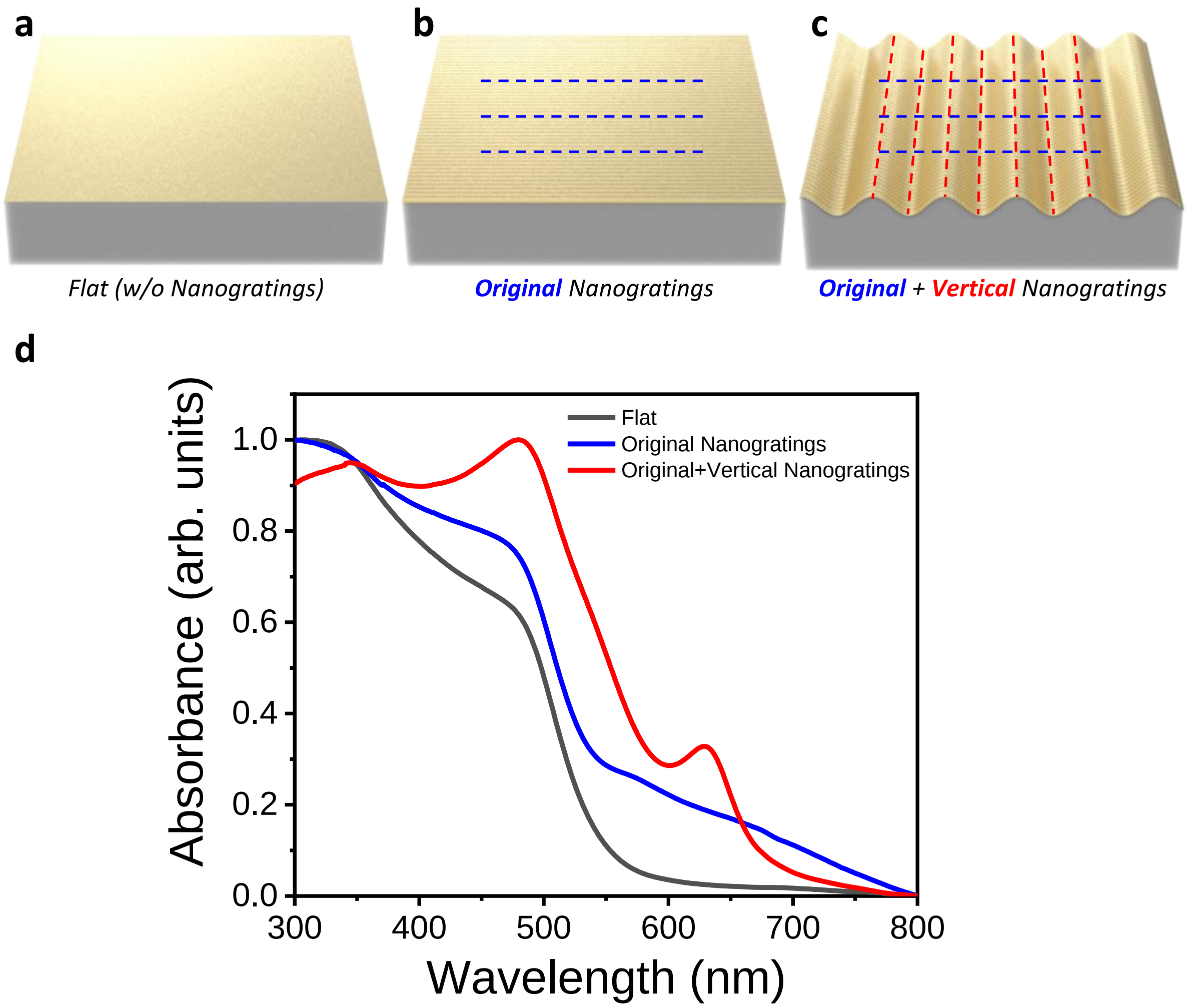

Supplementary Fig. 11| a-d, Schematic images of **(a)** flat PDMS/Au substrate, **(b)** PDMS/Au with the original nanogratings, **(c)** PDMS/Au substrate with the original nanogratings combined with the additionally formed vertical nanogratings, and **(d)** absorption spectra of those substrates.

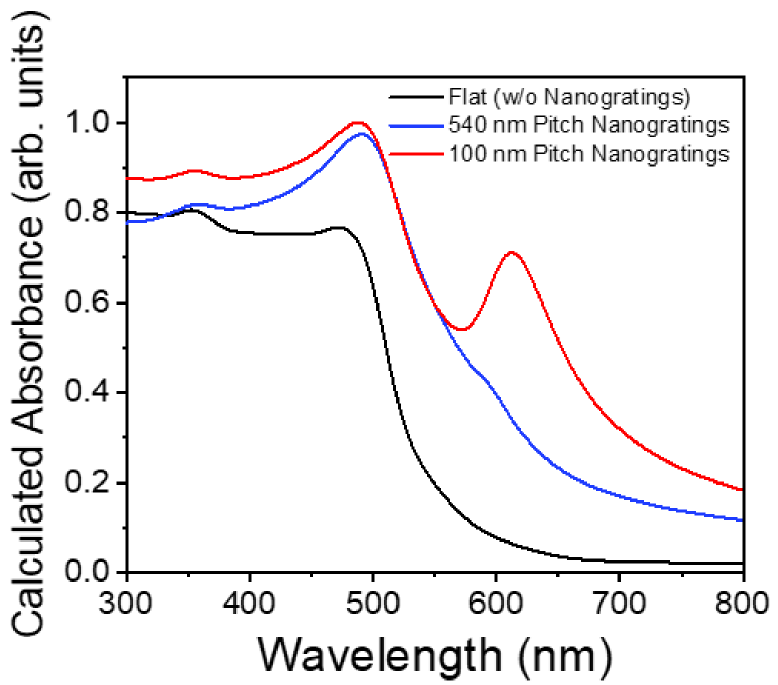

Supplementary Fig. 12| Calculated absorption spectra of flat PDMS/Au substrate, PDMS/Au substrate with the original nanogratings (540 nm pitch), and PDMS/Au substrate with the additionally formed nanogratings (100 nm pitch).

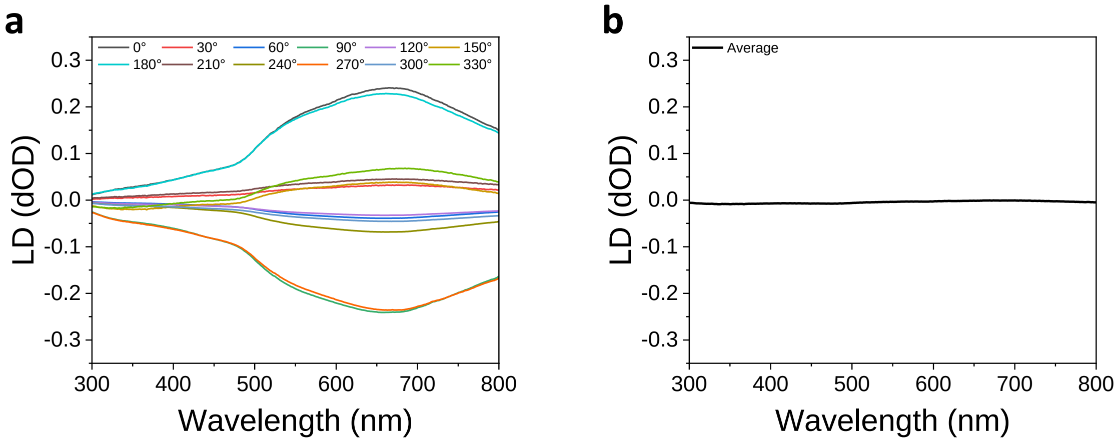

Supplementary Fig. 13| Linear dichroism spectra of LH-patterns. **(a)** Linear dichroism spectra of LH-patterns and **(b)** average LD value spectra of LH-patterns.

In DRCD measurement, the impact of linear dichroism (LD) caused by the optical anisotropy of a sample should be accounted for to minimize CD artifacts. LD refers to the difference between in absorption of light polarized parallel and perpendicular to an orientation axis. The effect of linear polarization on CD can be removed by measuring the sample at designated angular intervals and calculating the average value of the measured results. Our LD values at a 90° difference have opposite signs, indicating that the effect of LD can be removed from the average LH-pattern value based on a specific interval (30°).

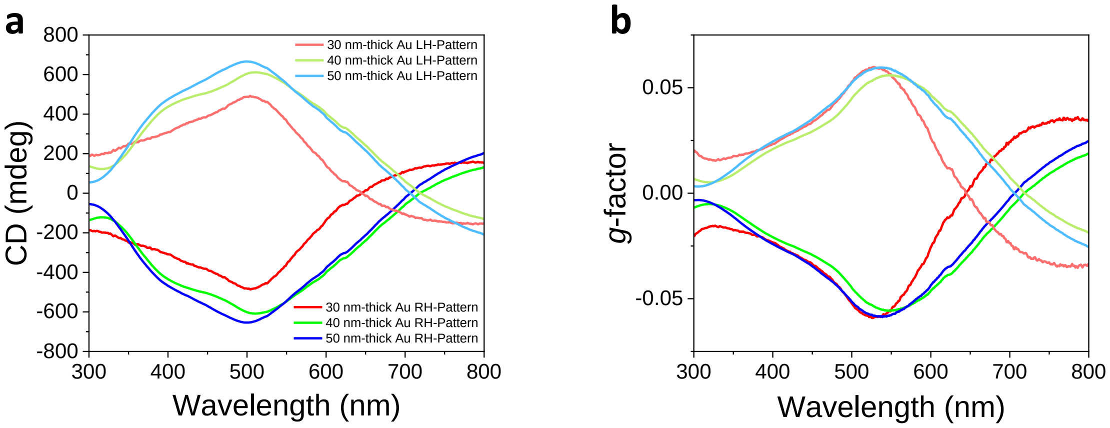

Supplementary Fig. 14| a,**b**, **(a)** CD and **(b)** *g*-factor spectra for LH- and RH-patterned chiral plasmonic PDMS films with an Au coating 30, 40, or 50 nm thick.

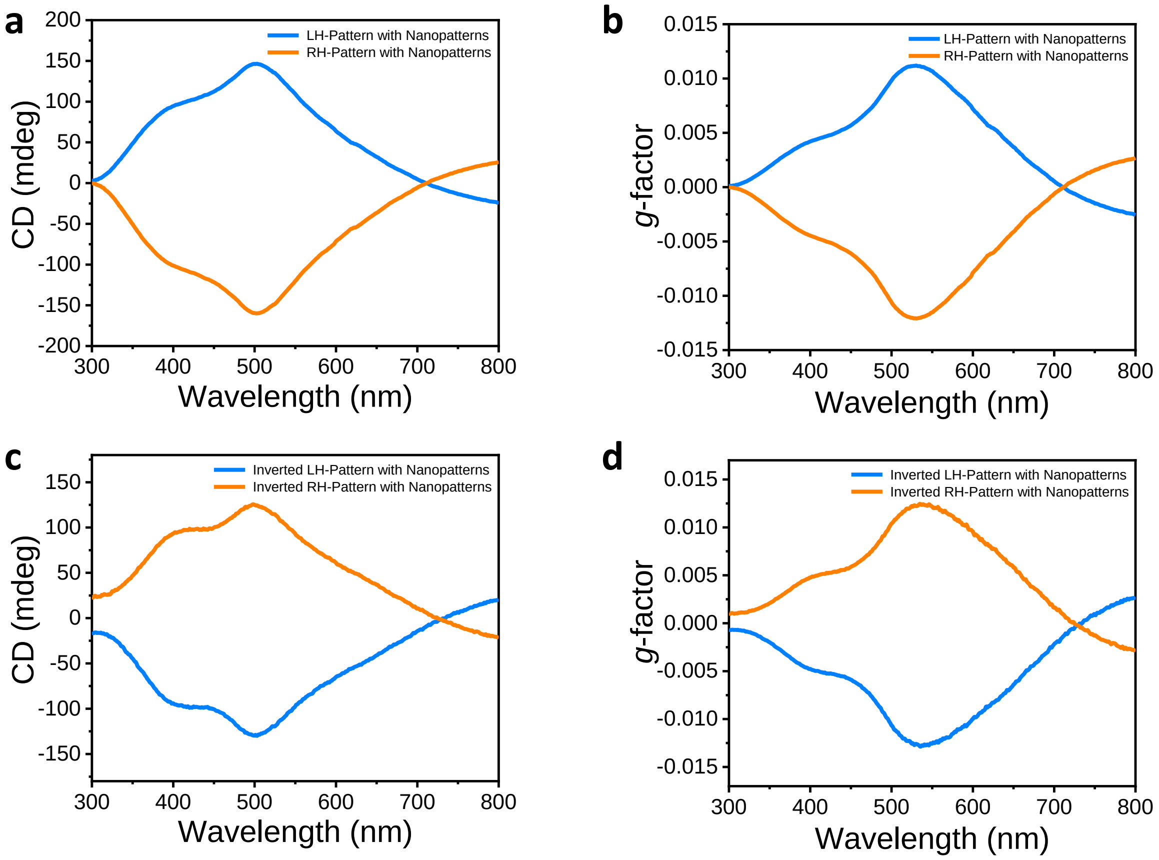

Supplementary Fig. 15| a,**b**, **(a)** CD and **(b)** *g*-factor spectra for the chiral plasmonic PDMS films. **c**,**d**, **(c)** CD and **(d)** *g*-factor spectra for the inverted LH- and RH-patterned chiral plasmonic PS films (Au thickness: 130 nm)

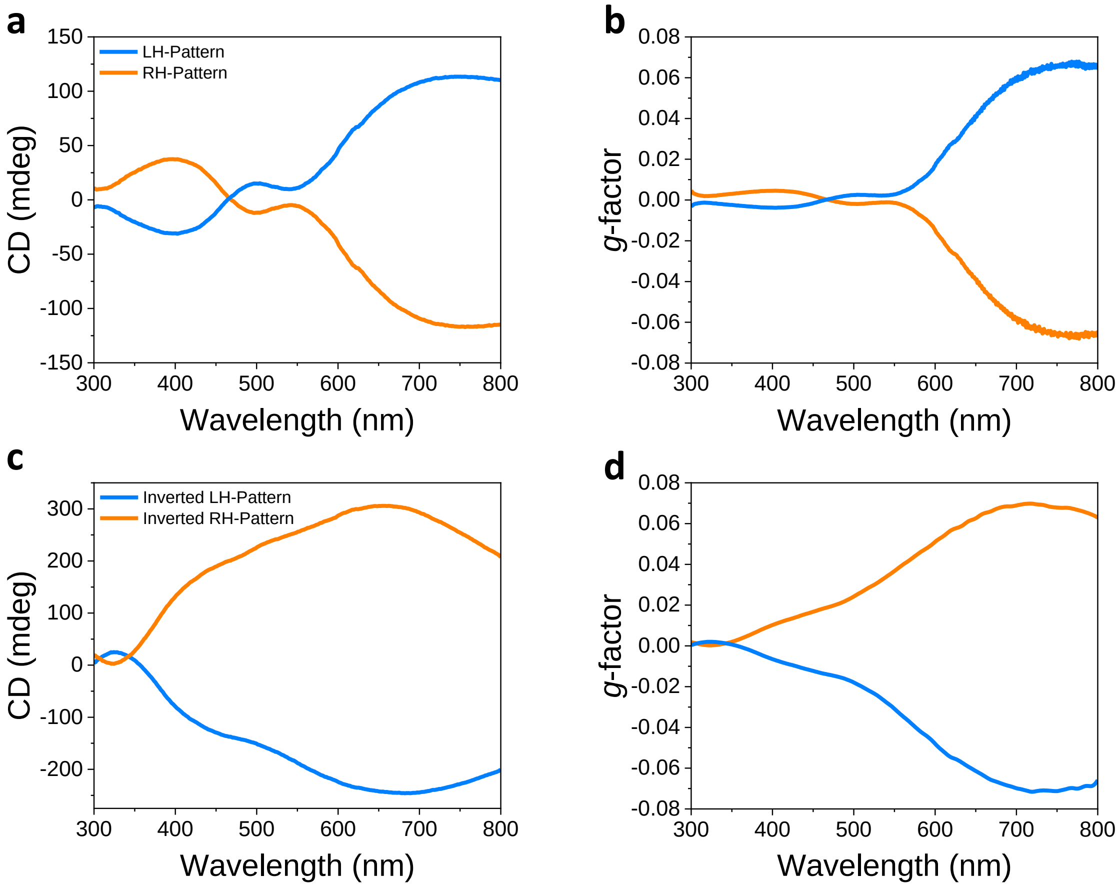

Supplementary Fig. 16| a,**b**, **(a)** CD and **(b)** *g*-factor spectra of LH- and RH-patterned chiral plasmonic PDMS films for the original nanograting patterns with a pitch size of 830 nm. **c**,**d,** **(c)** CD and **(d)** *g*-factor spectra of inverted-LH and RH-patterned chiral plasmonic PS films for the original nanograting patterns with a pitch size of 830 nm. (Au thickness: 40 nm)

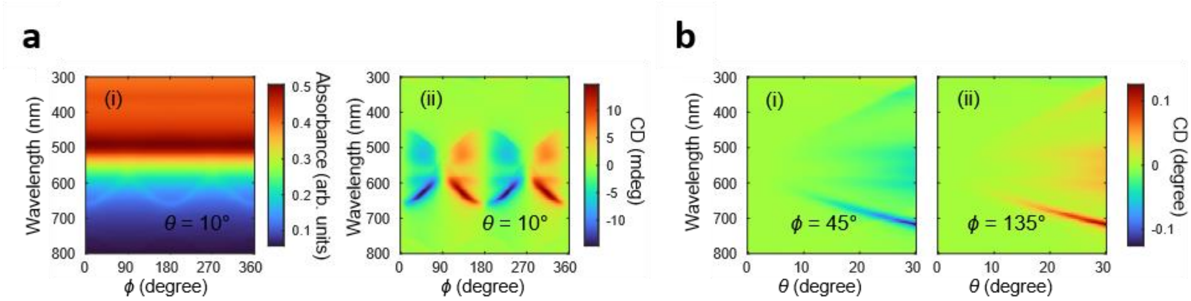

Supplementary Fig. 17| Numerically calculated obliquity angle dependence of optical characteristics for the original nanogratings. (a) (i) Absorbance and (ii) CD spectra of the diagonal nanogratings at the obliquity angle θ = 10° and different φ. **(b)** CD spectra of the diagonal nanogratings at the obliquity angles (i) φ = 45° and (ii) φ = 135° and different θ.

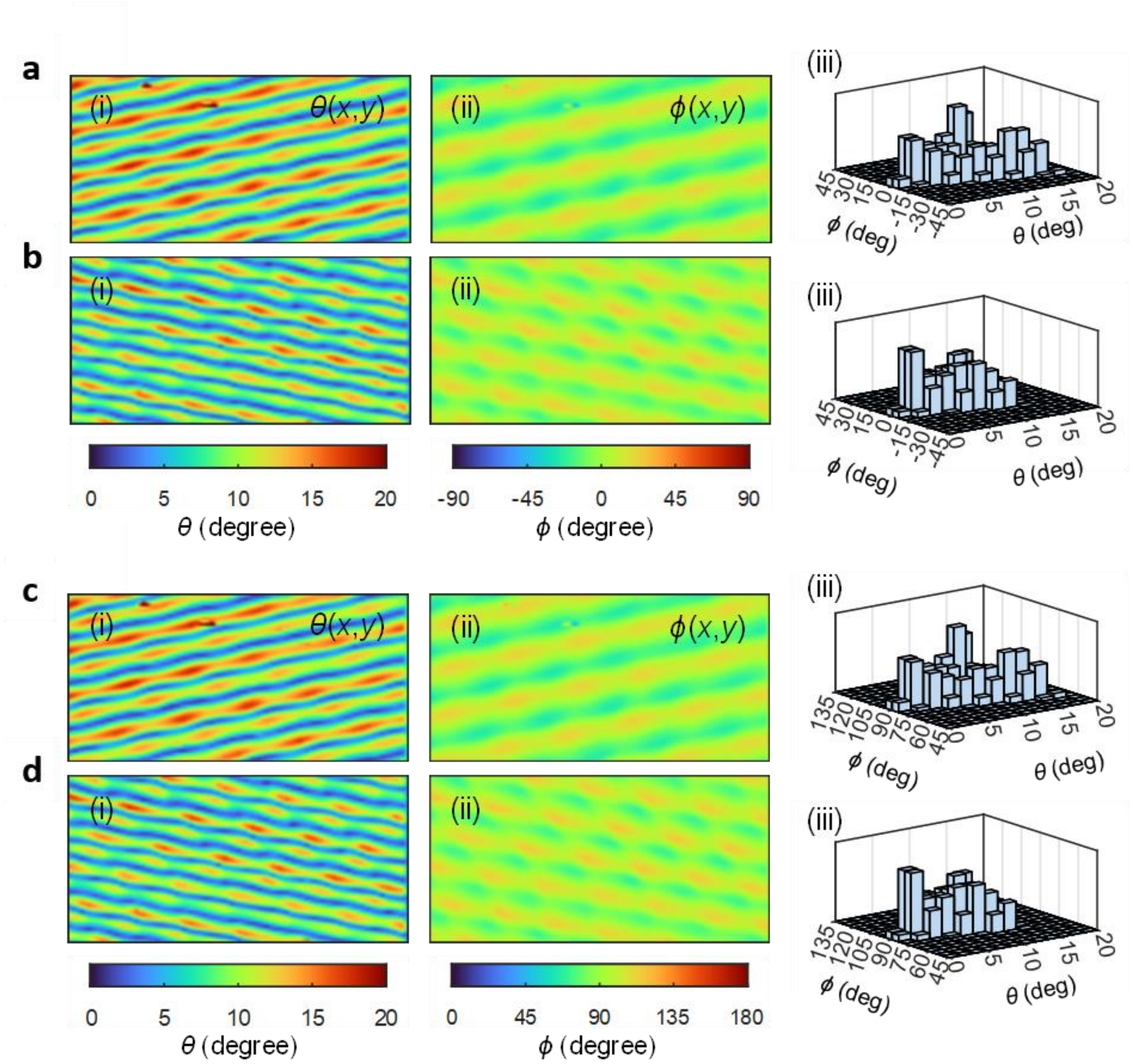

Supplementary Fig. 18| a-d, Macroscopic mapping of the θ (i) and ϕ (ii) results of the **(a, b)** vertical and **(c, d)** original nanopatterns from **(a, c)** LH- and **(b, d)** RH-patterned chiral plasmonic structures. (iii) Distribution histograms of the θ and ϕ results.

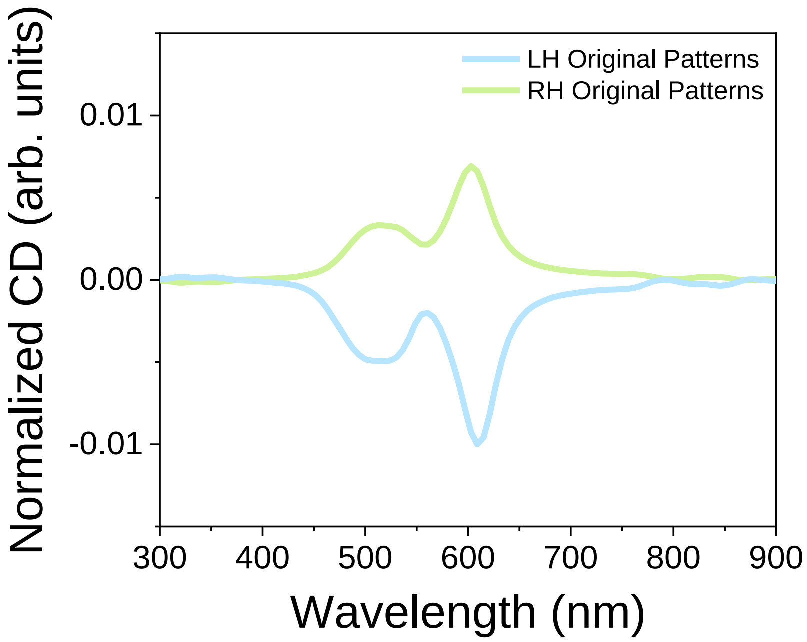

Supplementary Fig. 19| Calculated CD spectra of the chiral plasmonic structures for original nanograting patterns in Fig. 3d.

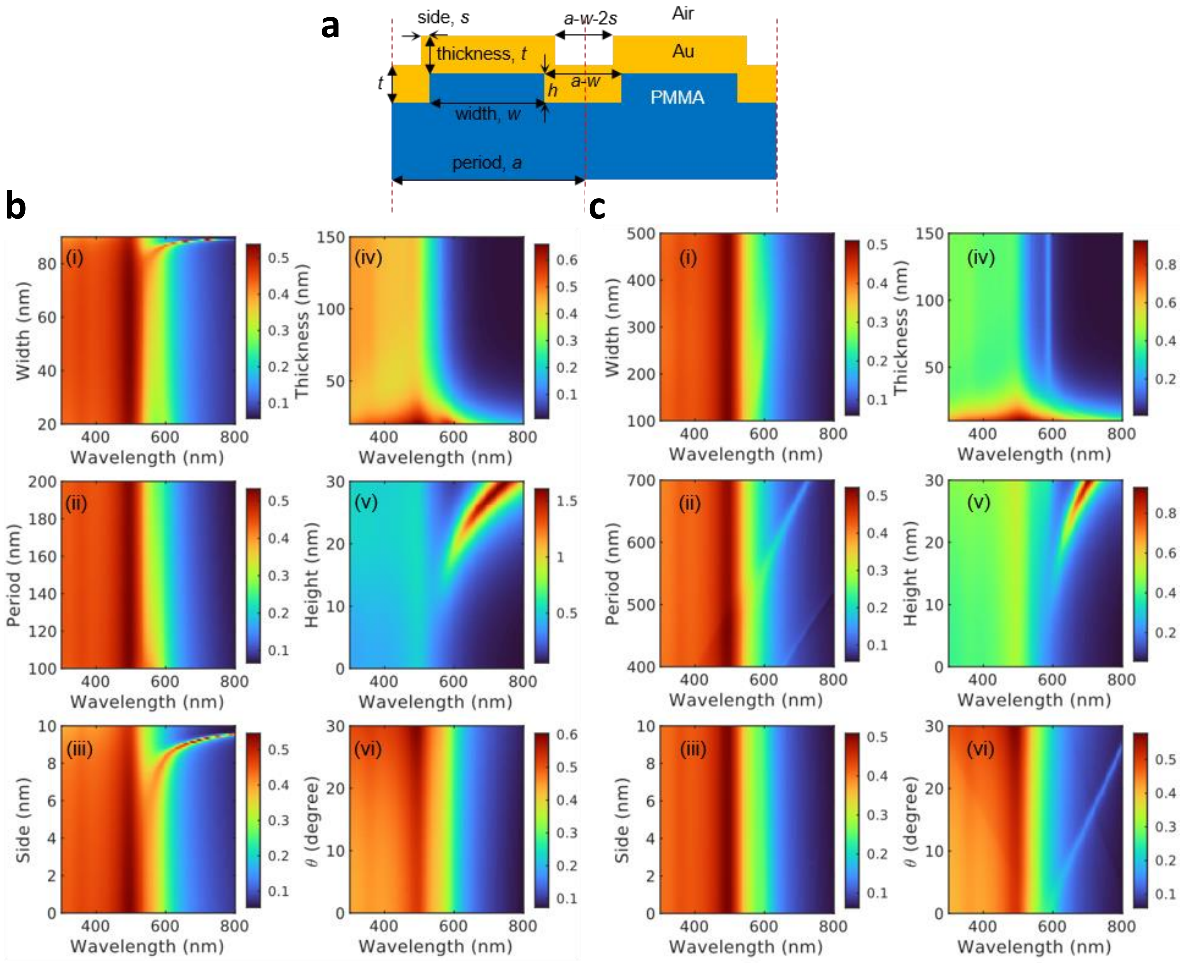

Supplementary Fig. 20| Parametric studies of nanogratings for different geometric parameters. **(a)** Schematic model of nanogratings. **b,c** Parametric studies of absorbance spectra by changing (i) width w, (ii) period a, (iii) Au side thickness s, (iv) Au thickness t, (v) grating height h, and (vi) incidence angle θ. The default parameters are: **(b)**, w = 80 nm, a = 100 nm, s = 5 nm, t = 30 nm, h = 10 nm, θ = 0°; and **(c)**, w = 300 nm, a = 540 nm, s = 5 nm, t = 30 nm, h = 10 nm, θ = 0°. The parametric studies were performed at p-polarized incident lights.

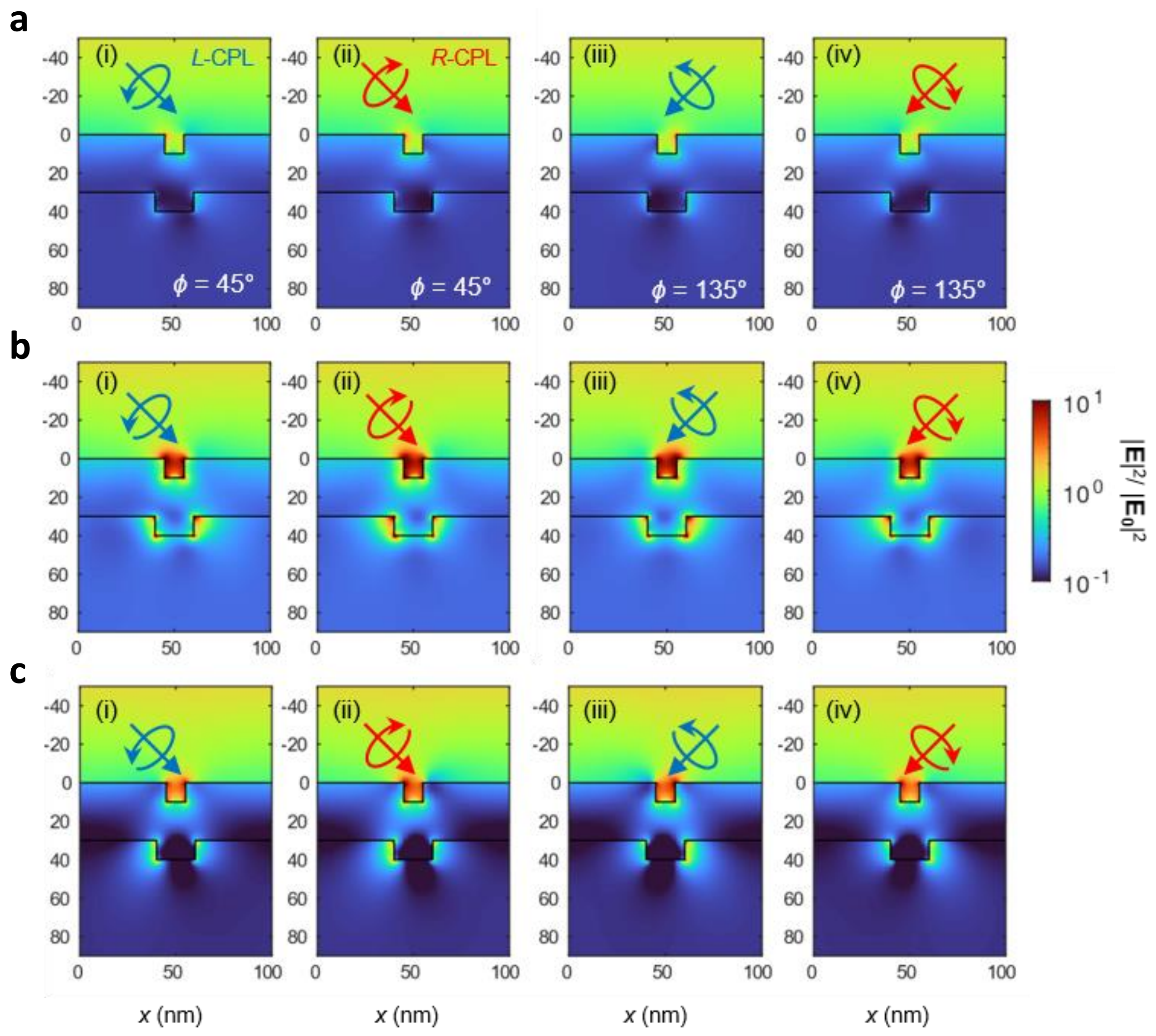

Supplementary Fig. 21| Near-field distribution of the diagonal nanogratings at RGB excitation wavelengths. a-c, The electric field intensity distribution at **(a)** 460 nm, **(b)** 543 nm, **(c)** 647 nm, under (i,iii) *L*-CPL and (ii,iv) *R*-CPL incident lights, (i,ii) φ = 45° and (iii,iv) φ = 135°. The calculations were performed at $\theta = 30°$.

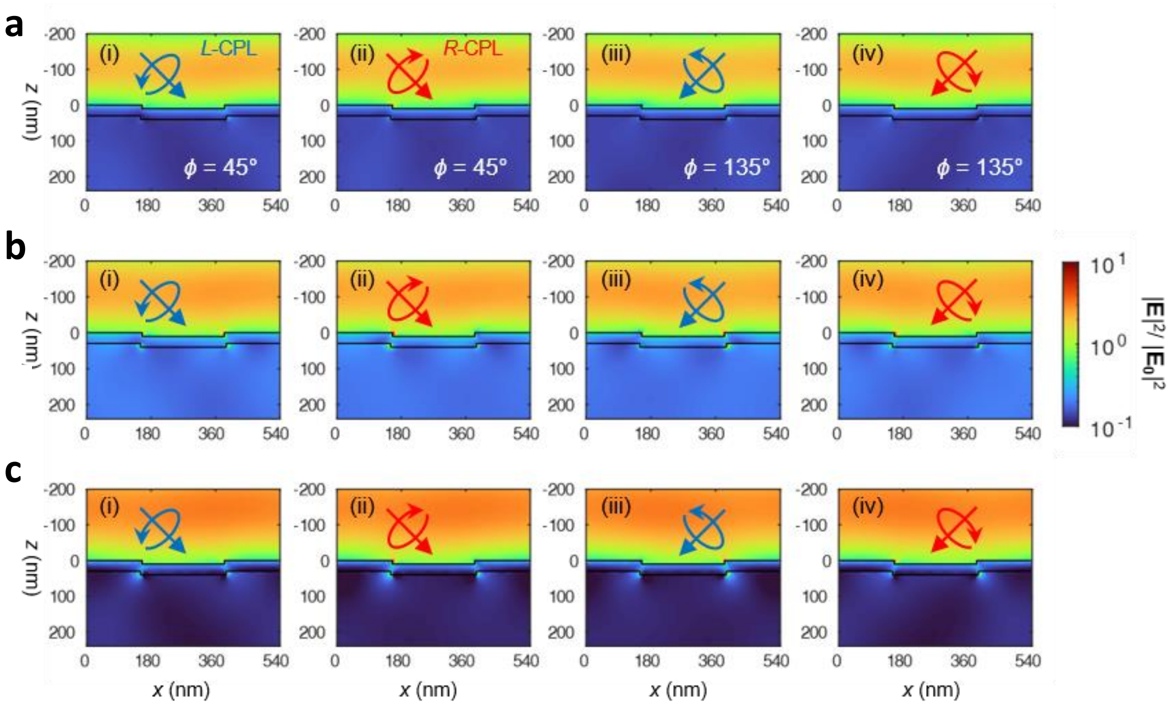

Supplementary Fig. 22| Near-field distribution of the original nanogratings at RGB excitation wavelengths. a-c, The electric field intensity distribution at **(a)** 460 nm, **(b)** 543 nm, **(c)** 647 nm, under (i,iii) *L*-CPL and (ii,iv) *R*-CPL incident lights, (i,ii) φ = 45° and (iii,iv) φ = 135°. The calculations were performed at θ = 30°.

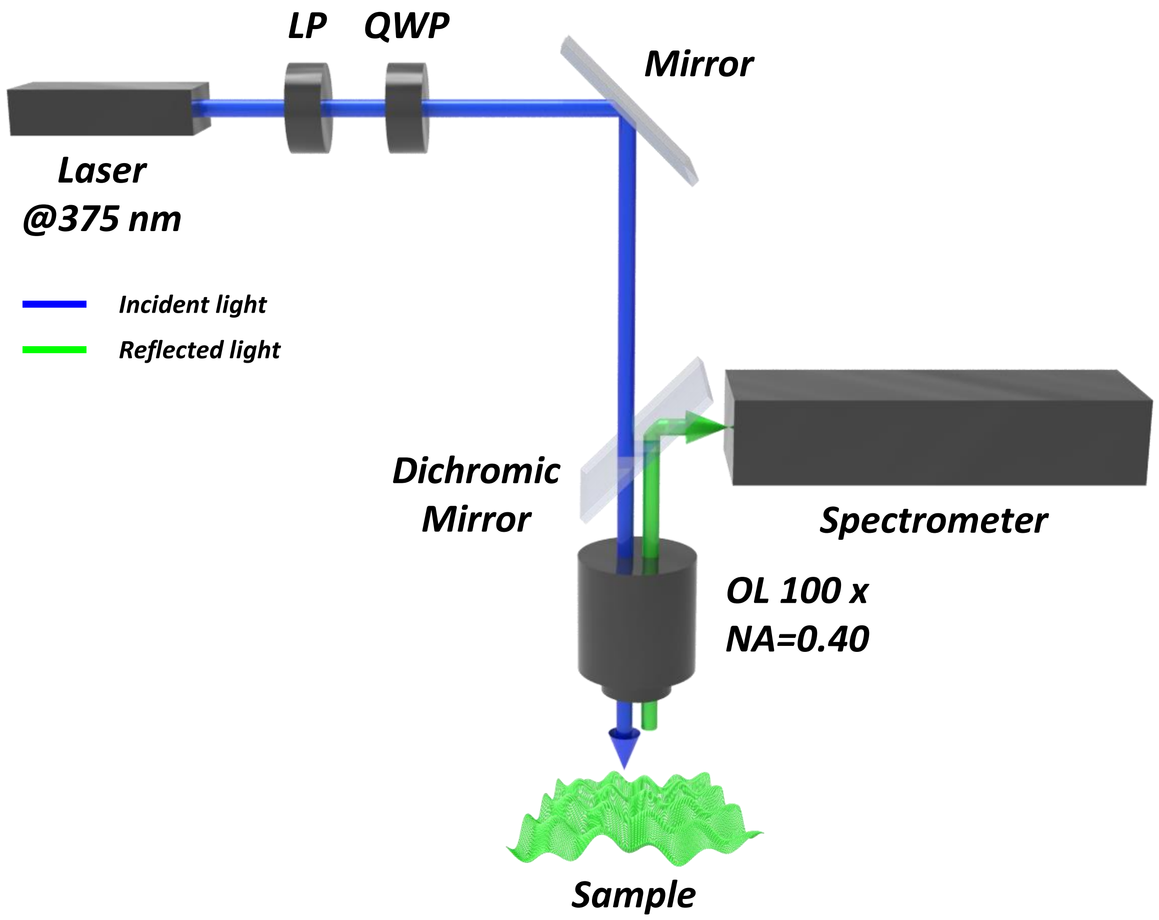

Supplementary Fig. 23| PL intensity measurement setup under CPL illumination (LP: linear polarizer, OL: objective lens, QWP: quarter wave plate, NA: numerical aperture).

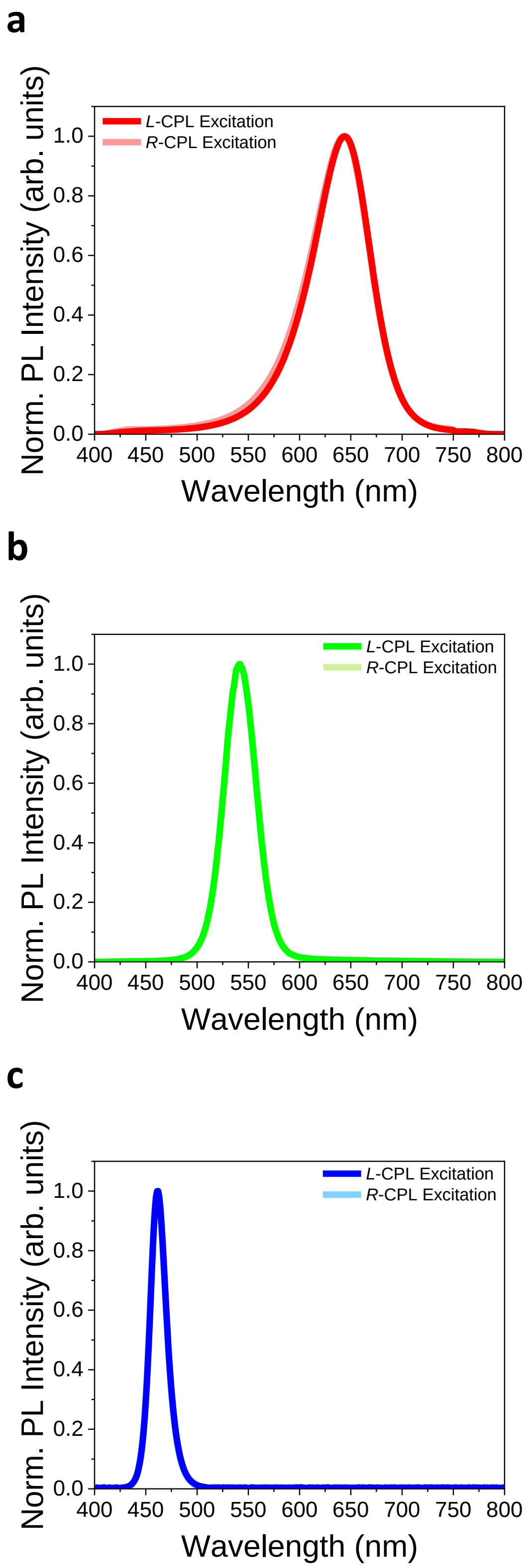

Supplementary Fig. 24| a-c, Polarization-non-sensitive PL spectra of **(a)** R-, **(b)** G-, and **(c)** B-QDs on flat gold substrates.

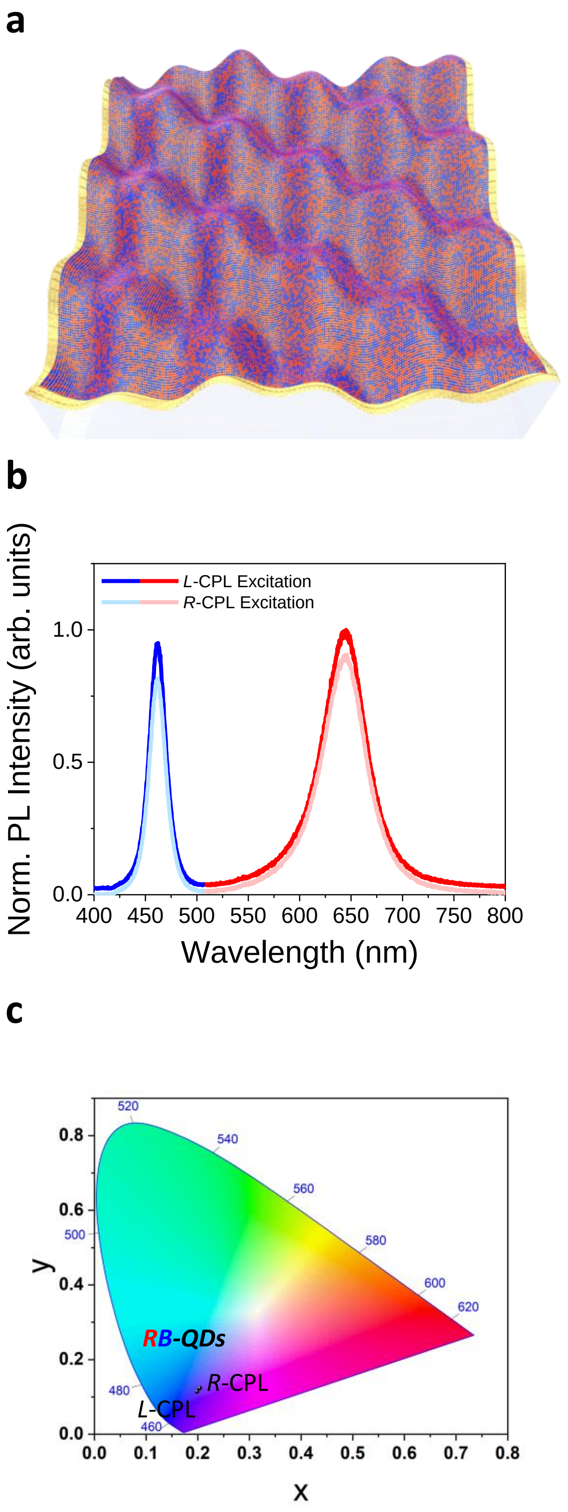

Supplementary Fig. 25| (a) Schematic image of RB-QD-coated RH-chiral plasmonic structures. **(b)** Polarization-sensitive PL spectra and schematic images of the RB-QD-based system. **(c)** CIE 1931 coordinates of the emitted light of the RB-QD-based system according to the CPL rotation direction.

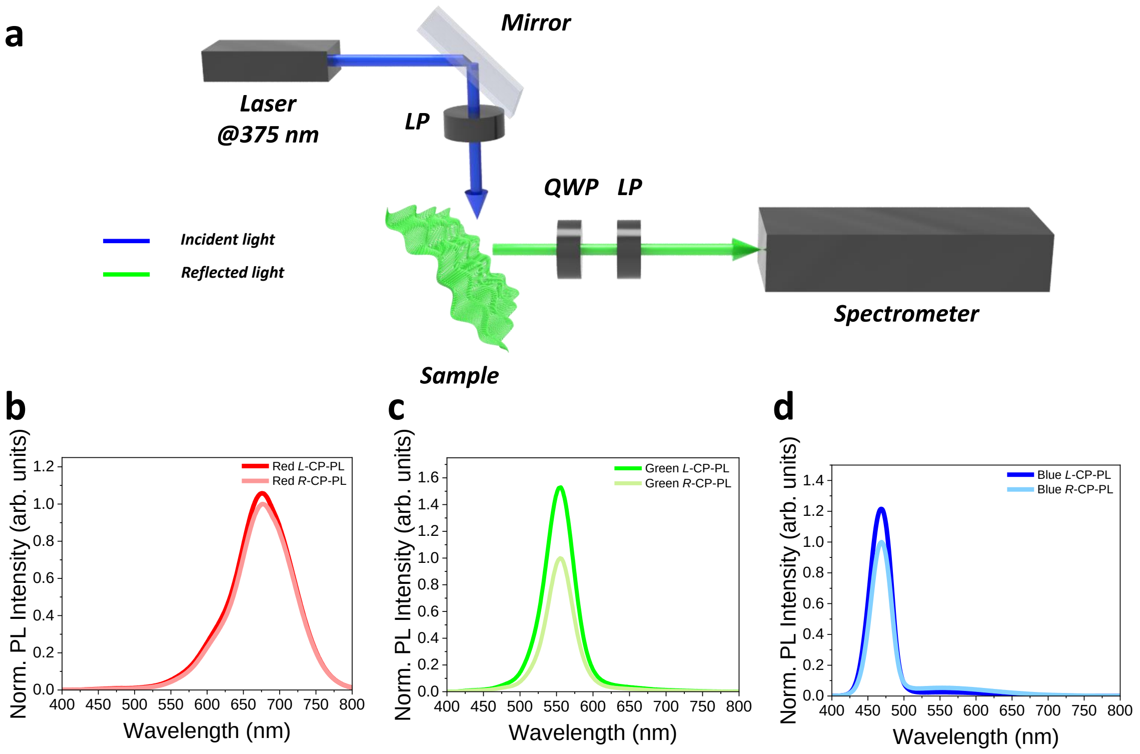

Supplementary Fig. 26| (a) PL-CPL measurement setup (LP: linear polarizer, QWP: quarter wave plate). **b-d**, The experimental circular polarized PL intensity of the **(b)** R-QDs, **(c)** G-QDs, and **(d)** B-QDs on the chiral plasmonic structures.

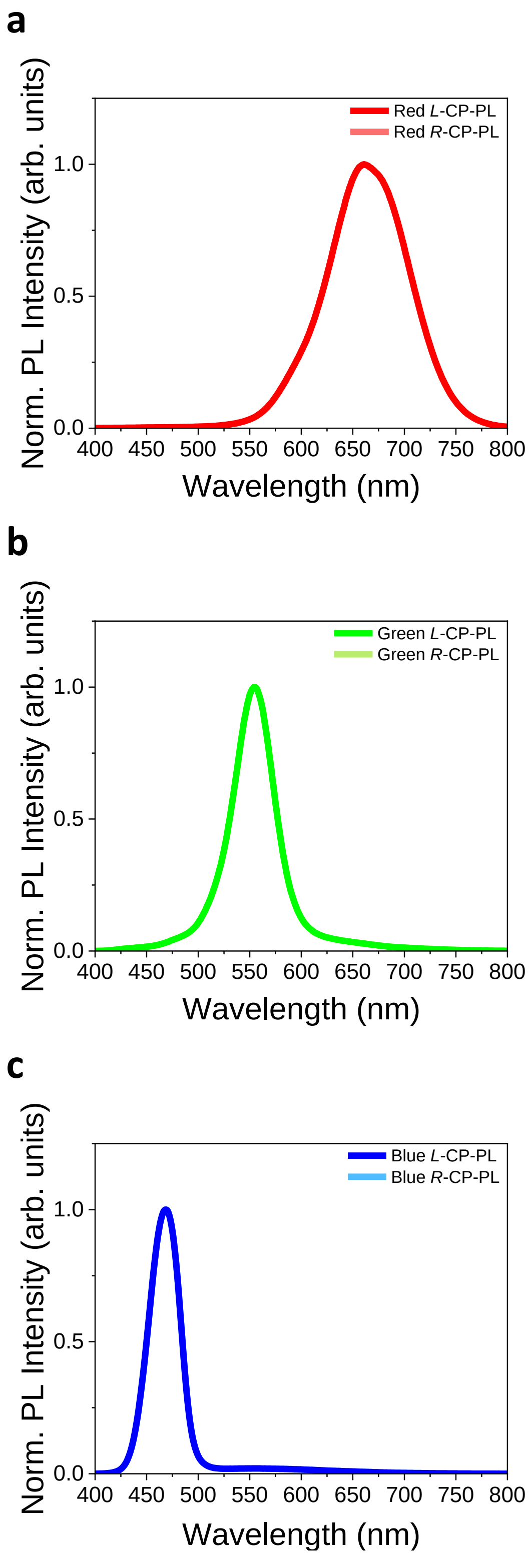

Supplementary Fig. 27| a-c, Polarization-non-sensitive PL spectra of **(a)** R-, **(b)** G-, and **(c)** B-QDs on flat gold substrates.

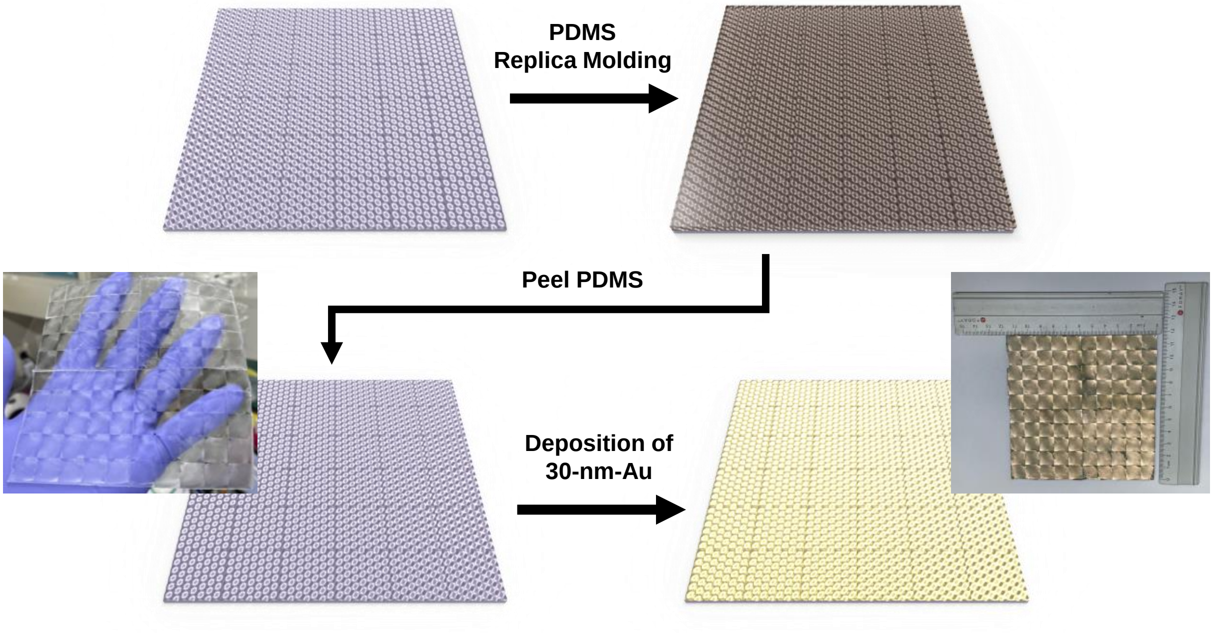

Supplementary Fig. 28| Schematic images of fabrication method of large-area chiral plasmonic patterns. (left inset: photograph image of large area chiral PDMS film, right inset: photograph image of large area chiral PDMS/Au film).

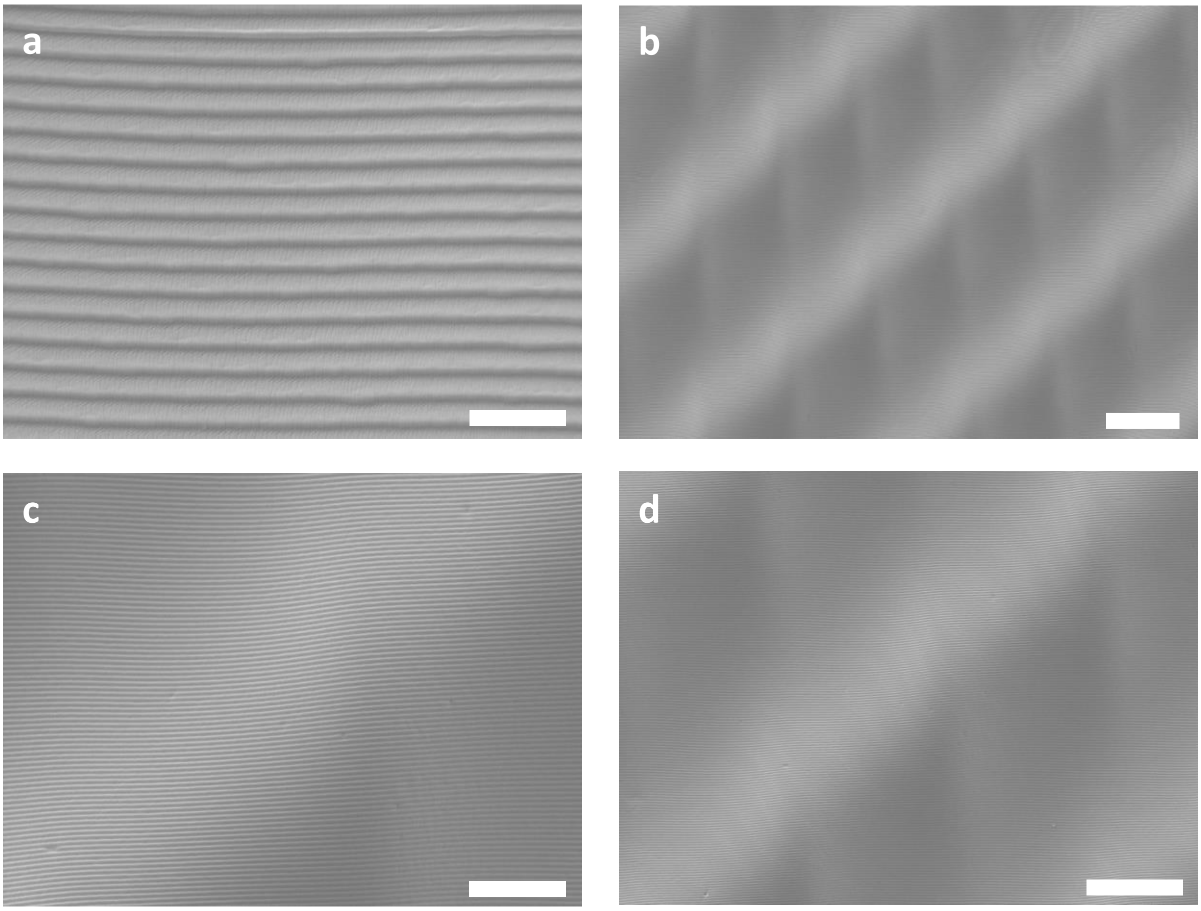

Supplementary Fig. 29| SEM images of inverted LH-patterned chiral plasmonic films based on PDMS substrates (scale bars of Supplementary Fig. 29a: 2 μm, 29b: 30 μm, 29c: 10 μm, 29d: 20 μm)

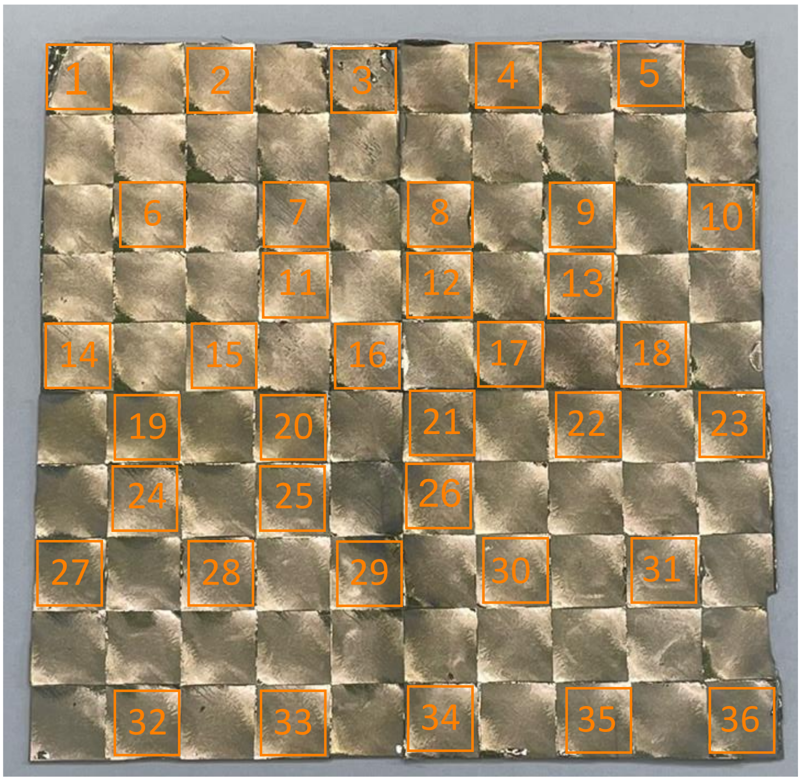

Supplementary Fig. 30| Schematic illustration of the fabricated 11.7 × 11.7 cm^2 chiral plasmonic film cut into 36 pieces of 1.3 cm by 1.3 cm films.

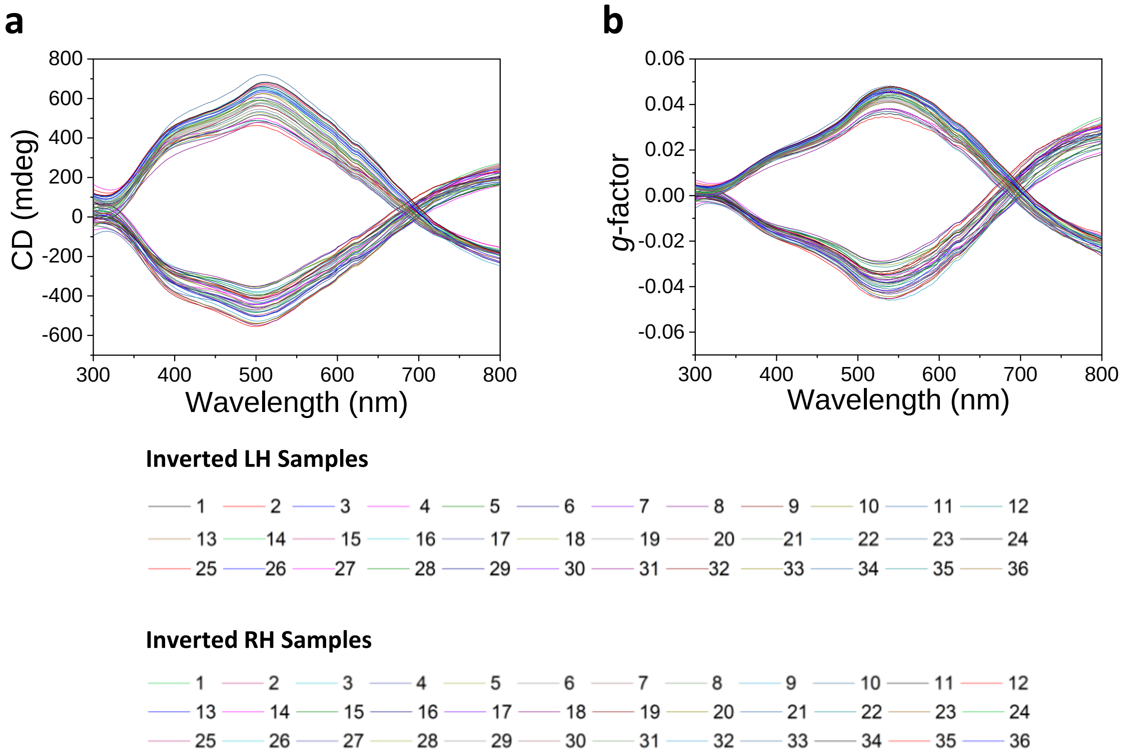

Supplementary Fig. 31| a,**b**, **(a)** CD and **(b)** *g*-factor spectra of the 36 pieces cut from a large area of the inverted LH- and RH- chiral plasmonic film samples.

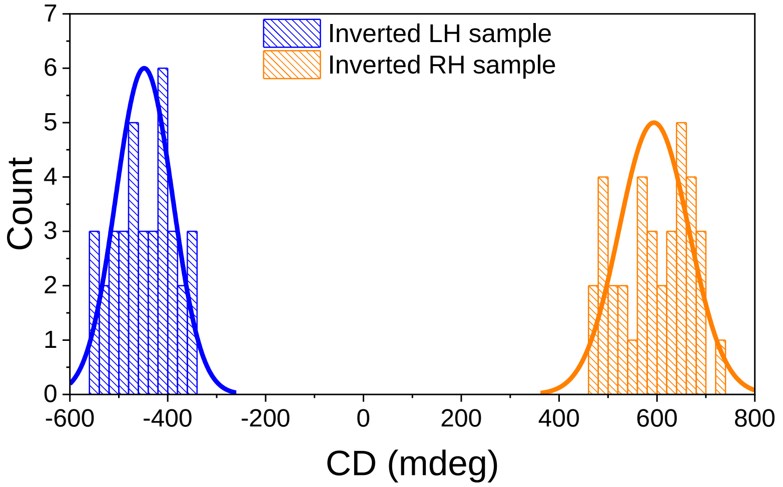

Supplementary Fig. 32| Maximum CD value distribution of the 36 pieces cut from large-area inverted LH- and RH-chiral plasmonic film samples.

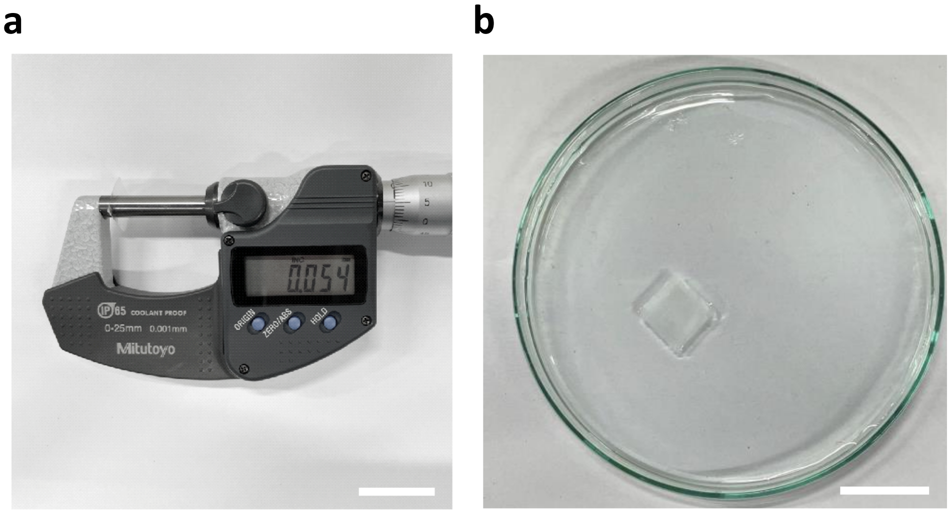

Supplementary Fig. 33| a,**b**, **(a)** Photograph of the chiral PDMS QR code film **(a)** for thickness measurement using micrometers (scale bar: 2 cm), and **(b)** the film floating in water (scale bar: 2 cm).

Supplementary Table 1. Comparison of the characteristics of chiral plasmonic structures reported in previous studies and in this work, in terms of material, approach, *g*-factor, area of structures, intrinsic/extrinsic chirality, and wavelength of light.

Material	Approach	g-factor$_{abs}$	Area (mm^2)	Intrinsic/Extrinsic	Wavelength (nm)	Ref.
Au	Particle	0.003	NA	Intrinsic	520	1
Au	Particle	0.019	NA	Intrinsic	530	2
Au	Particle	0.003	NA	Intrinsic	190	3
Au	Particle	0.0003	NA	Intrinsic	530	4
Ag	Particle	0.002	NA	Intrinsic	530	5
Au/Ag	Particle	0.01	NA	Intrinsic	430	6
Au	Particle	0.02	NA	Intrinsic	750	7
Au	Particle	0.03	NA	Intrinsic	700	8
Au	Particle	0.2	NA	Intrinsic	622	9
Au	Particle	0.024	NA	Intrinsic	600	10
Au	Particle	0.006	NA	Intrinsic	365	11
Au	Particle	0.4	NA	Intrinsic	600/710	12
PDMS/Au	FIB	NA	0.01	Intrinsic	1200~2800	13
ITO/Au	FIB	NA	0.0016	Intrinsic	3000~12000	14
Au/QDs	FIB	NA	0.0025	Intrinsic	450~700	15
Fused Silica/Au	E-Beam Lithography	NA	4	Intrinsic	600~2400	16
ITO/Au	E-Beam Lithography	NA	0.01	Intrinsic	1000~1800	17
SiO_2/Au	E-Beam Lithography	NA	0.064	Intrinsic	600~1400	18
Glass/Au/Dye	E-Beam Lithography	NA	0.04	Intrinsic	500~900	19
Ag/QDs	E-Beam Lithography	NA	625	Intrinsic	400~1000	20
ITO/PMMA/Au	E-Beam Lithography	NA	0.04	Intrinsic	526~833	21
Au/PMMA	GLAD with Hole Mask	NA	100	Intrinsic	750~3000	22
Au	GLAD with Low Temperaure		4560	Intrinsic	500~1000	23
Ag/SiO_2	GLAD with PS Sphere (4 times evaporation)	0.074	264	Intrinsic	614	24
Ag	GLAD with PS Sphere (2 times evaporation)	NA	100	Intrinsic	400~800	25
Au	GLAD with PS Sphere and RIE Etching	0.058	375	Intrinsic	600	26
Au	GLAD with PS sphere and RIE Etching	0.024	750	Intrinsic	600	27
Ti/Ag	GLAD with Room Temperature	0.39	NA	Intrinsic	500~520	28

PS/Ag	GLAD with PS Sphere<br>(2 times evaporation)	0.94	100	Intrinsic	700	29
PS/Ag	GLAD with PS Sphere and RIE Etching<br>(3 times evaporation)	0.07	1935	Intrinsic	664	30
Au NPs	Moiré<br>(Stacking/Compression)	0.12/0.05/0.72	625	Intrinsic	560/680/1290	31
Au/QDs	Moiré<br>(Stacking)	0.007	100	Intrinsic	608	32
PDMS/Au	Strain of Au Nanograting Structured Substrate	NA	625	Extrinsic	300~900	33
Parylene/Cr/Au	Strain	NA	150	Extrinsic	0.2~1.6 THz	34
PDMS/Au NPs	Strain/Twisting Au NPs Coated Substrate	0.025	300	Extrinsic	620	35
PDMS/Au	Stretching of Substrate with BCP Lithography & Metallization	0.0089	625	Extrinsic	680	36
PDMS/Au, PS/Au	Stretching of Au Nanograting Structured Substrate	0.056/0.07	13689	Extrinsic	557/700	This Work

- **FIB**: focused ion beam
- **GLAD**: glancing angle deposition
- **RIE**: reactive ion etching
- **PMMA**: polymethylmethacrylate
- **BCP**: block-copolymer
- **ITO**: indium tin oxide
- **NPs**: nanoparticles

Supplementary References

1 Wu, X., *et al.* Unexpected Chirality of Nanoparticle Dimers and Ultrasensitive Chiroplasmonic Bioanalysis. *J. Am. Chem. Soc*. **135**, 18629-18636 (2013).

2 Yan, W., *et al.* Self-Assembly of Chiral Nanoparticle Pyramids with Strong R/S Optical Activity. *J. Am. Chem. Soc.* **134**, 15114-15121 (2012).

3 Gautier, C., Bürgi, T. Chiral N-Isobutyryl-cysteine Protected Gold Nanoparticles: Preparation, Size Selection, and Optical Activity in the UV−vis and Infrared. *J. Am. Chem. Soc.* **128**, 11079-11087 (2006).

4 Slocik, J. M., *et al.* Plasmonic Circular Dichroism of Peptide-Functionalized Gold Nanoparticles. *Nano Lett.* **11**, 701-705 (2011).

5 Maoz, B. M., *et al.* Plasmonic Chiroptical Response of Silver Nanoparticles Interacting with Chiral Supramolecular Assemblies. *J. Am. Chem. Soc.* **134**, 17807-17813 (2012).

6 Hao, C., *et al.* Unusual Circularly Polarized Photocatalytic Activity in Nanogapped Gold–Silver Chiroplasmonic Nanostructures. *Adv. Funct. Mater.* **25**, 5816-5822 (2015).

7 Wu, X., *et al.* Propeller-Like Nanorod-Upconversion Nanoparticle Assemblies with Intense Chiroptical Activity and Luminescence Enhancement in Aqueous Phase. *Adv. Mater.* **28**, 5907-5915 (2016).

8 Kuzyk, A., *et al.* DNA-based self-assembly of chiral plasmonic nanostructures with tailored optical response. *Nature* **483**, 311-314 (2012).

9 Lee, H.-E., *et al.* Amino-acid- and peptide-directed synthesis of chiral plasmonic gold nanoparticles. *Nature* **556**, 360-365 (2018).

10 Lee, H.-E., *et al.* Cysteine-encoded chirality evolution in plasmonic rhombic dodecahedral gold nanoparticles. *Nat. Commun.* **11**, 263 (2020).

11 George, J., *et al.* Chiral Plasmons: Au Nanoparticle Assemblies on Thermoresponsive Organic Templates. *ACS Nano* **13**, 4392-4401 (2019).

12 Xu, L., *et al.* Enantiomer-dependent immunological response to chiral nanoparticles. *Nature* **601**, 366-373 (2022).

13 Liu, Z., *et al.* Fano-Enhanced Circular Dichroism in Deformable Stereo Metasurfaces. *Adv. Mater.* **32**, 1907077 (2020).

14 Gansel, J. K., *et al.* Gold Helix Photonic Metamaterial as Broadband Circular Polarizer. *Science* **325**, 1513-1515 (2009).

15 Wang, Z., *et al.* Induced Optical Chirality and Circularly Polarized Emission from Achiral CdSe/ZnS Quantum Dots via Resonantly Coupling with Plasmonic Chiral Metasurfaces. *Laser Photonics Rev.* **13**, 1800276 (2019).

16 Dietrich, K., *et al.* Circular Dichroism from Chiral Nanomaterial Fabricated by On-Edge Lithography. *Adv. Mater.* **24**, OP321-OP325 (2012).

17 Decker, M., *et al.* Strong optical activity from twisted-cross photonic metamaterials. *Opt. Lett.* **34**, 2501-2503 (2009).

18 Zhao, Y., *et al.* Twisted optical metamaterials for planarized ultrathin broadband circular polarizers. *Nat. Commun.* **3**, 870 (2012).

19 Le, K. Q., *et al.* Circularly Polarized Photoluminescence from Achiral Dye Molecules Induced by Plasmonic Two-Dimensional Chiral Nanostructures. *J. Phys. Chem. C* **122**, 24924-24932 (2018).

20 Rodrigues, S. P., *et al.* Metamaterials Enable Chiral-Selective Enhancement of Two-Photon Luminescence from Quantum Emitters. *Adv. Mater.* **27**, 1124-1130 (2015).

21 Zhang, R., *et al.* Measuring circular phase-dichroism of chiral metasurface. *Nanophotonics* **8**, 909-920 (2019).

22 Frank, B., *et al.* Large-Area 3D Chiral Plasmonic Structures. *ACS Nano* **7**, 6321-6329 (2013).

23 Mark, A. G., *et al.* Hybrid nanocolloids with programmed three-dimensional shape and material composition. *Nat. Mater.* **12**, 802-807 (2013).

24 Ullah, H., *et al.* Tunable chiroptical response of chiral system composed of a nanorod coupled with a nanosurface. *Appl. Surf. Sci.* **467**, 684-690 (2019).

25 Hou, Y., *et al.* Ultrabroadband Optical Superchirality in a 3D Stacked-Patch Plasmonic Metamaterial Designed by Two-Step Glancing Angle Deposition. *Adv. Funct. Mater.* **26**, 7807-7816 (2016).

26 Guan, Y., *et al.* Chiral Plasmonic Metamaterials with Tunable Chirality. *ACS Appl. Mater. Interfaces* **12**, 50192-50202 (2020).

27 Wang, Z., *et al.* Free-Standing Plasmonic Chiral Metamaterials with 3D Resonance Cavities. *ACS Nano* **12**, 10914-10923 (2018).

28 Larsen, G. K., *et al.* Scalable Fabrication of Composite Ti/Ag Plasmonic Helices: Controlling Morphology and Optical Activity by Tailoring Material Properties. *Adv. Opt. Mater.* **2**, 245-249 (2014).

29 Tang, C., *et al.* Large-area cavity-enhanced 3D chiral metamaterials based on the angle-dependent deposition technique. *Nanoscale* **12**, 9162-9170 (2020).

30 Ai, B., *et al.* Chiral nanohole arrays. *Nanoscale* **12**, 2479-2491 (2020).

31 Probst, P. T., *et al.* Mechano-tunable chiral metasurfaces via colloidal assembly. *Nat. Mater.* **20**, 1024-1028 (2021).

32 Aftenieva, O., *et al.* Tunable Circular Dichroism by Photoluminescent Moiré Gratings. *Adv. Opt. Mater.* **9**, 2001280 (2021).

33 Jeong, K.-J., *et al.* Chirality of Fingerprints: Pattern- and Curvature-Induced Emerging Chiroptical Properties of Elastomeric Grating Meta-Skin. *ACS Nano* **16**, 6103-6110 (2022).

34 Choi, W. J., *et al.* Terahertz circular dichroism spectroscopy of biomaterials enabled by kirigami polarization modulators. *Nat. Mater.* **18**, 820-826 (2019).

35 Kim, Y., *et al.* Reconfigurable chiroptical nanocomposites with chirality transfer from the macro- to the nanoscale. *Nat. Mater.* **15**, 461-468 (2016).

36 Cho, J., *et al.* Chiral Plasmonic Nanowaves by Tilted Assembly of Unidirectionally Aligned Block Copolymers with Buckling-Induced Microwrinkles. *ACS Nano* **15**, 17463-17471 (2021).
